# A Removable Mechanical Interface for Non-Invasive Strain Measurement in Paddle Shafts: Iterative Design and Experimental Characterization

**DOI:** 10.3390/s26196220

**Published:** 2026-09-30

**Authors:** Maria I. Cruz, Beatriz B. Gomes, Marco Silva, Ana M. Amaro, Luis Roseiro

**Affiliations:** 1 CIPER, Faculty of Sport Sciences and Physical Education, University of Coimbra, 3040-248 Coimbra, Portugal; beatrizgomes@fcdef.uc.pt; 2 CEMMPRE-ARISE, Department of Mechanical Engineering, University of Coimbra, 3030-788 Coimbra, Portugal; ana.amaro@dem.uc.pt (A.M.A.); lroseiro@isec.pt (L.R.); 3 Coimbra Institute of Engineering, Polytechnic University of Coimbra, 3030-199 Coimbra, Portugal; msilva@isec.pt; 4 Research Centre for Natural Resources Environment and Society (CERNAS), Polytechnic University of Coimbra, 3045-601 Coimbra, Portugal

**Keywords:** kayak, canoeing, paddle instrumentation, strain gauge, contact mechanics, sports engineering, fused filament fabrication

## Abstract

Accurate force measurement on paddle shafts is essential for performance assessment and technique refinement, yet existing tools typically require permanent shaft modification or recalibration after each utilization. This study presents the iterative design and characterization of a removable, non-invasive interface for strain measurement on cylindrical paddle shafts, designed for repeated installation without bonding. A total of nineteen configurations were developed through an iterative five-phase design process using fused filament fabrication (FFF) and evaluated under static and dynamic laboratory loading, including an exploratory on-water assessment of the distributed-clamping shell with one athlete. Performance was benchmarked against a bonded reference using linearity, hysteresis, repeatability, and signal fidelity. Rigid shells improved linearity but retained high hysteresis, while distributed clamping introduced placement-dependent uncertainty. Beam-based geometries reduced contact sensitivity, and the distal gauge position of an inverted U-shaped beam provided the closest agreement with the reference response under the tested conditions, reaching a coefficient of determination of 0.954–0.990, a mean absolute error of 7.8–17.7 N, and hysteresis of 3.4–9.1% of the applied load range. Its residual hysteresis was concentrated in the low-force unloading region, predominantly near the transition from the propulsive to the recovery phase. No configuration eliminated all trade-offs; acceptable performance depended on the intended measurement context. The resulting framework provides testable design indications for the future development of removable strain-sensing interfaces on cylindrical structures.

## 1. Introduction

In paddle-based water sports such as sprint kayaking and canoeing, the shaft is the primary structural element through which the athlete transmits propulsive force to the paddle blade and, ultimately, to the water, making it the natural measurand location for force instrumentation. Quantifying these forces under on-water training and competition conditions is essential for performance assessment, technique optimization, and the development of evidence-based training programs [1,2]. The magnitude and temporal profile of the paddle are strongly associated with kayak velocity and paddling efficiency [3,4,5], making accurate measurement of these parameters a central objective of sports biomechanics instrumentation. These force profiles exhibit rapid loading and unloading within each stroke, along with inter-limb differences in force magnitude [2], placing demanding requirements on the temporal fidelity and accuracy of instrumentation design to capture these dynamics.

The dominant approach for measuring force in paddle shafts employs resistive strain gauges bonded directly to the shaft surface and configured in Wheatstone bridge circuits to detect bending-induced surface deformation [6,7]. This method offers high measurement sensitivity, inappreciable added mass, wide frequency response, and straightforward signal conditioning [8]. It has been used extensively to characterize force profiles across different stroke rates [3,4], quantify propulsive forces during on-water sprint efforts [5,9], and investigate inter-paddler force asymmetry in crew boats [2]. However, adhesively bonded configurations require irreversible surface preparation and adhesive application, permanently modifying the equipment and precluding its reuse across different users, experimental protocols, or competition paddles.

More broadly, existing instrumentation solutions can be classified into three distinct levels of invasiveness: the first and most prevalent category comprises systems in which sensing elements are adhesively bonded to the outer shaft surface [1,2,3,4,9,10]; the second category includes solutions requiring structural modification of the shaft itself, such as replacing a shaft section with a purpose-built instrumented segment [11]; the third category encompasses systems that require access to the hollow shaft interior, either during manufacturing or through existing mechanical adjustment features. An approach that is impractical for paddles lacking an adjustment zone, where access requires removing the blades, an intervention unacceptable to athletes for whom even minor equipment changes alter the feel of the stroke [12,13]. To the best of the authors’ knowledge, no system currently published/developed simultaneously avoids all three categories while maintaining measurement performance comparable to bonded configurations. This gap is not explicitly identified in the most recent comprehensive review of kayak instrumentation systems [13]. That review addresses transmission technology, sampling strategies, and sensor fusion rather than the mechanical fidelity of strain transmission through removable interfaces, confirming that the contact mechanics of non-invasive sensing assemblies remain unaddressed in the literature.

Several commercial and research systems have attempted to reduce invasiveness with varying degrees of success. The Trainesense SmartPaddle^®^ provides stroke metrics without shaft modification, but it systematically overestimates peak forces relative to bonded strain-gauge references [14]. Reported correlation coefficients range from 0.70 to 0.95, depending on the variable considered [14]. The One Giant Leap™ Kayak Power Meter integrates sensing elements within a purpose-built fiber shaft [11]. Among research prototypes, Petrone et al. reported an early load acquisition device for Olympic kayak shafts [9]. That work did not characterize the fidelity of deformation transmission through the sensing interface. The mechanical factors governing strain transfer between the shaft and the sensor were left unaddressed, thereby directly motivating the present study. More recently, Sturm et al. [15] developed a wireless paddle sensor network that uses a U-shaped aluminum bracket and a screw to achieve surface contact with the shaft. Without recalibration after each installation, the average measurement error rises from 0.4% to about 2.0%. This reveals a fundamental dependence on the calibration state [15]. Guseinov et al. subsequently developed a removable sensor assembly based on a curved metal beam fixed to the shaft with clamps, cable ties, and an antifriction layer [16]. Good linearity (Pearson r ≈ 1.00) and repeatability were demonstrated for the optimal fixation method. However, the system exhibited nonlinearity below approximately 10 N, sensitivity to clamping torque, and installation-dependent recalibration requirements arising from positional variability. Critically, neither study systematically investigated the mechanical factors governing the transmission of deformation through the beam–shaft interface, leaving the contact mechanics of removable sensing assemblies largely unexplored.

To address these requirements, there remains a need for a removable instrumentation system that fulfills two key requirements. It should approximate the measurement response of a bonded strain-gauge configuration under defined loading and mounting conditions, while allowing removable installation without permanent shaft modification. Shell interfaces fabricated by fused filament fabrication (FFF) represent a promising manufacturing approach. They offer conformal geometry, design flexibility, and cost-effective reproduction [17,18]. However, introducing a contact interface between the shaft and the sensor raises non-trivial mechanical challenges. Strain transmission fidelity depends on interface compliance, contact pressure distribution, and micro-slip behavior under cyclic loading. These factors are well established in the contact mechanics literature [19,20,21] but have not been systematically characterized in the context of removable paddle instrumentation interfaces.

This study aims to design, fabricate, and characterize a removable mechanical shell-type interface for instrumenting paddle shafts with strain gauges. The interface is intended for repeated use in both laboratory and field environments without permanent modification. The specific objectives are: (i) to design and fabricate a conformal FFF interface and determine whether, and under which mounting and sensor-position conditions, it can approximate the measurement response of a bonded configuration; (ii) to characterize the strain transmission behavior of the interface under static and dynamic loading; and (iii) to characterize the measurement accuracy and test–retest repeatability of the complete instrumented assembly against a bonded reference system.

## 2. Materials and Methods

The removable interface was developed according to four main functional requirements: (i) installation without permanent modification of the shaft, (ii) preservation of sufficient strain-transmission fidelity to resolve the characteristic force profile of a paddle stroke, (iii) repeatable installation under laboratory and field conditions, and (iv) compatibility with subsequent use on different paddles and users. The last requirement represents a long-term design objective; transferability across different shaft geometries and athletes was not evaluated in the present study. These requirements are inherently competing because the contact layer required for removability introduces compliance, contact-pressure variability, and possible relative displacement that are absent from a directly bonded strain gauge.

**Figure 1:**
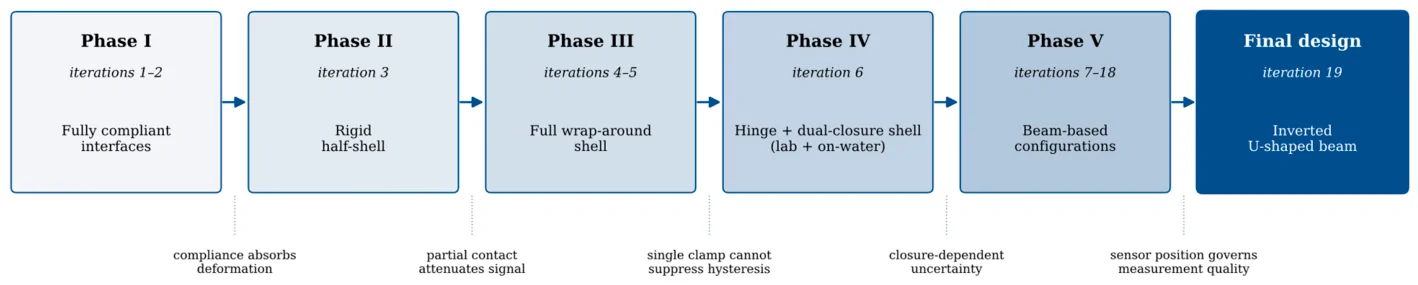
Overview of the iterative development methodology: nineteen configurations organized into five conceptual phases, culminating in the evaluation of the inverted U-shaped beam and its distal sensing position (iteration 19).

The development of the removable interface followed an iterative design methodology comprising nineteen configurations organized into five conceptual phases, as illustrated in Figure 1. Phase I (iterations 1–2) explored fully compliant interfaces; Phase II (iteration 3) introduced a rigid half-shell; Phase III (iterations 4–5) extended the concept to full circumferential enclosure; Phase IV (iteration 6) added a full-length continuous hinge with a dual closure system, evaluated both in the laboratory and on water; and Phase V (iterations 7–19) shifted to beam-based configurations, culminating in the evaluation of the inverted U-shaped beam and the selection of its distal sensing position as the basis for subsequent development. Each phase was defined by a distinct structural concept, and transitions between phases were triggered by the identification of structural, rather than parametric, limitations, as described in Section 2. This section presents the materials, instrumentation, and evaluation methods common to all phases; phase-specific protocols are described in the corresponding subsections of Section 3.

Because the development process was exploratory, absolute quantitative acceptance thresholds were not defined a priori. Each configuration was compared with the bonded reference and with the preceding configuration using the performance indicators available at the corresponding development stage. The bonded reference was treated as a performance benchmark rather than as an equivalence criterion.

Design decisions were based on the persistence and apparent origin of the dominant limitation. A limitation was classified as parametric when its magnitude or expression changed meaningfully following adjustments to dimensions, material, sensor position, fixation, or other design parameters without changing the underlying load-transfer mechanism. Parametric limitations motivated further optimization within the same structural concept. In contrast, a limitation was classified as structural when it persisted across the tested parameter variations or boundary conditions and was associated with the underlying load path, contact topology, closure mechanism, or sensor-integration strategy. Structural limitations motivated the transition to a different design concept.

The decision process comprised four steps: (i) identification of the dominant limitation from the available fabrication, static, dynamic, or field evidence; (ii) assessment of whether the limitation responded to feasible modifications within the current concept; (iii) classification of the limitation as parametric or structural; and (iv) continuation of within-concept optimization or transition to a new structural concept, respectively. The evidence supporting each major transition is summarized in Table 1. Because metrics such as the coefficient of determination (R^2^), hysteresis, mean absolute error (MAE), and root mean square error (RMSE) do not have universally accepted acceptance thresholds, the configuration selection in this study was based on comparative performance rather than on normative cut-off criteria.

All interface configurations were manufactured using fused filament fabrication (FFF), enabling rapid design iteration and low-cost production. The primary structural material was poly(ethylene terephthalate glycol) (PETG; Prusament PETG, Prusa Research, Prague, Czech Republic), selected for its stiffness, dimensional stability, and compatibility with the FFF process. All components were printed on an Original Prusa XL (Prusa Research, Prague, Czech Republic). Thermoplastic polyurethane (TPU 82A, Filaflex, Recreus, Elda, Spain) was used in the compliant configurations of Phase I, and dental silicone (Reline II Extra Soft, GC, Tokyo, Japan) was used for the molded compliant interface.

Two types of strain gauges were used, depending on the bridge configuration. The quarter-bridge configurations used HBM 1-LY48-6/350V3 gauges (Hottinger Baldwin Messtechnik, Darmstadt, Germany), with a nominal resistance of 350 Ω and a gauge factor of 2.05 ± 1.0%. In contrast, the full-bridge configurations used BF350-3EB (Youmile, Shenzhen, China), with a nominal resistance of 350 Ω and a gauge factor of 2.15 ± 10.0%. In all configurations, the gauges were positioned along the interface’s longitudinal axis and aligned with the shaft’s expected bending direction. Within each configuration, the same gauge model was used simultaneously on the shell interface and on the shaft as the bonded reference, ensuring that both signals were acquired under identical electrical conditions. All surface-mounted gauges used in the quantitative evaluation were installed by the same operator following a consistent procedure comprising light surface abrasion, treatment with HBM acidic and neutralizing surface-conditioning agents, and bonding with a cyanoacrylate adhesive. Gauge alignment was controlled using the marks incorporated into the gauge backing. Gauge resistance was verified before and after bonding, and signal stability was confirmed before experimental testing.

**Figure 2:**
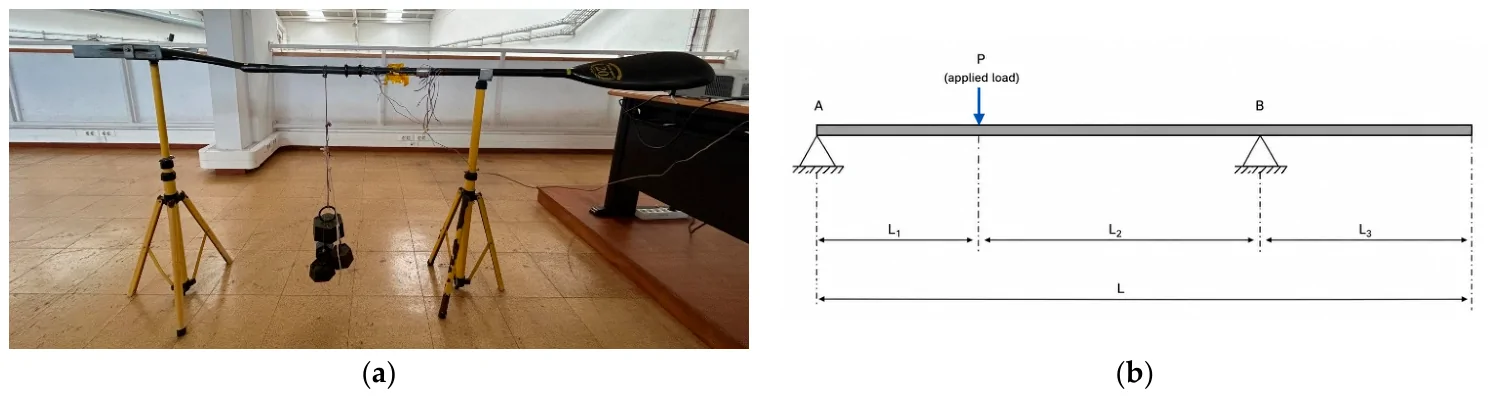
Static test setup: (**a**) Photograph of the experimental setup used for the static bending test, with the paddle shaft supported at the blade and top-hand positions and subjected to incremental loading at the bottom-hand position using calibrated weights. (**b**) Schematic representation of the experimental configuration, showing the support locations, the applied point load (P), the distances (L_1_, L_2_, and L_3_), and the total beam length (L).

Data acquisition was performed using two systems, depending on the development phase. For Phases I–IV (iterations 1–6), a V-Link wireless acquisition system (MicroStrain, Williston, VT, USA) was used at a sampling frequency of 256 Hz. For Phase V (iterations 7–19), a custom-developed PCB incorporating an AD7124-8 24-bit sigma-delta analog-to-digital converter (ADC) (Analog Devices, Wilmington, MA, USA) was used at a sampling frequency of approximately 70 Hz, corresponding to a temporal resolution of approximately 14.3 ms. All signals were processed in MATLAB (R2023b; MathWorks, Natick, MA, USA). This sampling frequency is consistent with those reported for portable on-water kayak instrumentation systems, in which acquisition rates are selected by balancing temporal resolution with data storage, wireless transmission, and energy-consumption constraints [13,16]. In the present study, the controlled dynamic tests were intended to compare the removable configurations under identical acquisition conditions, focusing on overall waveform agreement, peak magnitude, repeatability, and hysteresis rather than on impact-type events or high-frequency transients. This acquisition frequency was used consistently throughout this development phase. However, because no signal-spectrum analysis was performed, its adequacy for resolving sharp load transitions or higher-frequency transient components was not established. The dynamic comparisons should therefore be interpreted as evaluations of the overall waveform and cycle-level response rather than as validation of high-frequency force-pulse reconstruction. Across the quantitatively evaluated phases, performance was assessed by comparing measurements with a reference obtained from the same shaft under identical loading conditions. In the standard static configuration (Figure 2), the shaft was supported on two tripods at the blade and top-hand positions and loaded incrementally at the bottom-hand position, located 500 mm from the blade-side support, using calibrated weights covering the expected operating range; load ranges and protocol variations specific to each phase are described in the corresponding sections.

As the design evolved, the experimental protocol and the associated evaluation metrics were refined accordingly. Table 2 summarizes, for each phase, the acquisition system, bridge configuration, force reference, and reported metrics, thereby establishing the provenance of every value presented in Section 3. This progressive enrichment reflects the protocol’s increasing maturity rather than an inconsistency in the evaluation approach. Accordingly, cross-phase quantitative comparisons in this article rely exclusively on the coefficient of determination (R^2^), for the iterations in which this metric was available; the remaining metrics characterize each phase within its own acquisition chain and are not compared across phases.

For the on-water evaluation of Phase IV (Section 3.4), four cycle-level metrics were computed for each sensing channel: residual slope (slopeRes), absolute mid-point offset (|ΔMid|), proportionality ratio (R), and symmetry index (S). These metrics complement the laboratory evaluation metrics but are not mathematically equivalent to them. In the controlled laboratory tests, R^2^, MAE, and RMSE quantified agreement with a reference response, while hysteresis quantified the difference between the loading and unloading paths. Under on-water conditions, the absence of a known externally applied force prevented these metrics from being interpreted as measures of absolute measurement accuracy. Therefore, slopeRes and |ΔMid| were used to characterize cycle-dependent drift and baseline stability, respectively; R assessed gain proportionality relative to the selected reference signal; and S assessed inter-channel symmetry. Together, these metrics evaluate signal stability and internal consistency under field conditions. All metrics were computed cycle-by-cycle and summarized using the median and interquartile range; their formal definitions are provided in Appendix A (Table A1).

## 3. Design Evolution

This section describes the experimental evolution of the removable interface across the five conceptual phases introduced in Section 2 (Figure 1). Each subsection presents one phase, following a common structure: the design rationale motivating the configuration, its experimental evaluation, and the observations that exposed the limitation driving the transition to the next phase. Results are reported using the metrics and acquisition chains summarized in Table 2, with cross-phase comparisons restricted to R^2^, as established in Section 2. The section closes with the beam-based configurations of Phase V, from which the configuration selected for subsequent development emerged (iteration 19).

### 3.1. Phase I—Fully Compliant Interfaces

**Figure 3:**
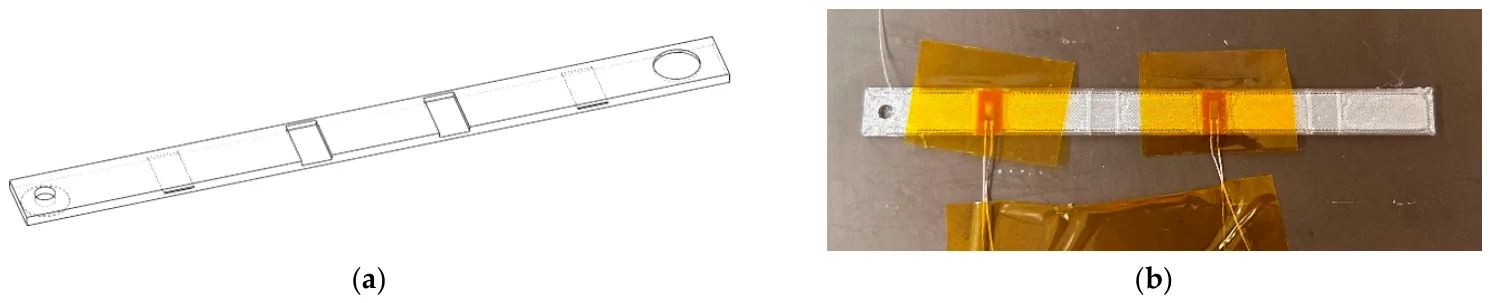
TPU elastic sleeve (iteration 1): (**a**) CAD model and (**b**) real prototype.

The first concept was a fully elastic TPU sleeve mounted on the shaft via circumferential pretension, with the inner diameter set slightly below the shaft diameter (28 mm) to ensure continuous contact through elastic deformation (Figure 3a). The sleeve, approximately 105 mm long and 1.5 mm thick, was manufactured by FFF with a mid-print pause introduced to embed the strain gauge within the structure (Figure 3b).

**Figure 4:**
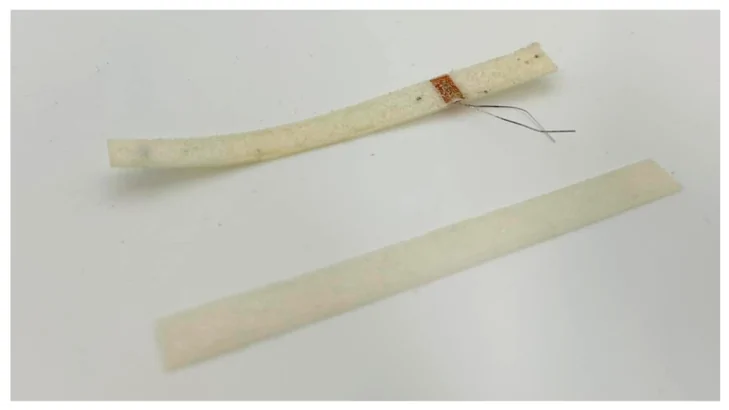
Interlayer delamination visually observed near the embedded strain-gauge region of the TPU sleeve.

Visual inspection revealed interlayer delamination around the embedded gauge (Figure 4). The spatial association between the defect and the embedded-gauge region suggested that the mid-print pause may have contributed to local disruption of interlayer bonding. Exploratory adjustments to the printing conditions, including reduced print speed and the use of a heated build plate, did not eliminate the visually observed defect. However, no microscopy or dedicated interlayer adhesion tests were performed to confirm the underlying failure mechanism. Consequently, reliable mechanical testing of this configuration could not be conducted.

**Figure 5:**
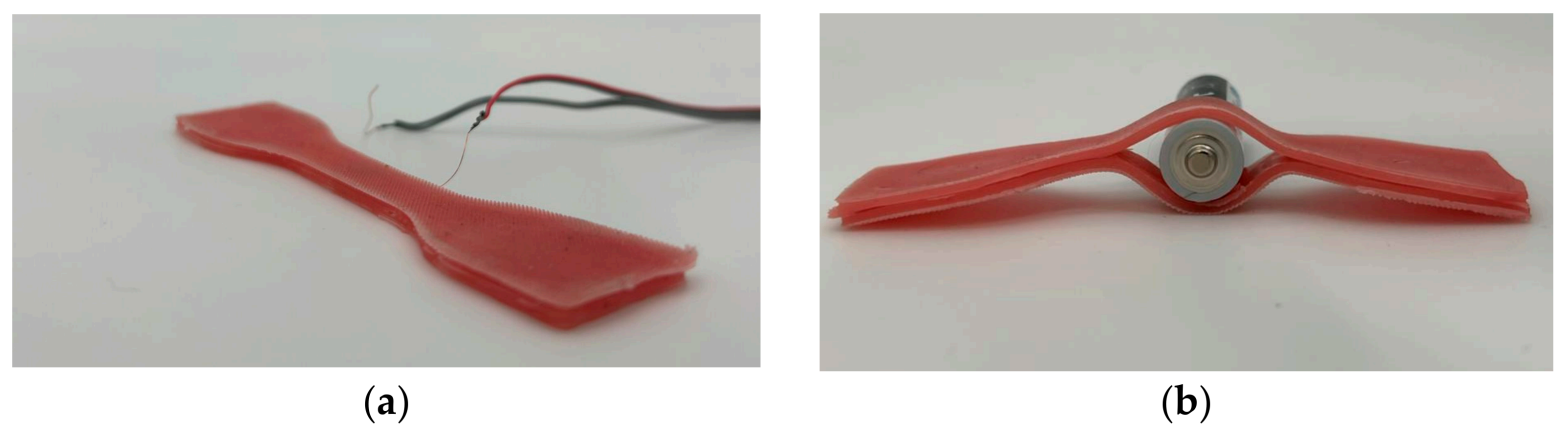
Dental silicone interface (iteration 2): (**a**) final specimen after curing; (**b**) local detachment between the embedded strain gauge and the surrounding silicone developed during experimental evaluation.

The second concept exploited the natural conformability and casting capability of dental silicone: the interface was cast in a 3D-printed two-part mold, with the strain gauge positioned between successive silicone layers to fully encapsulate the sensor. After curing, the specimen exhibited excellent geometric conformity and surface finish (Figure 5a). During experimental evaluation, however, local detachment was visually observed between the gauge and the surrounding silicone (Figure 5b). The low surface energy of the cured silicone and the stiffness difference between the gauge backing and the compliant matrix were considered possible contributors to this behavior. However, no microscopy, dedicated adhesion testing, or quantitative interfacial characterization was performed, and the specific detachment mechanism could therefore not be confirmed. The observed detachment compromised signal integrity before systematic data collection could be completed.

Both compliant configurations exhibited visually observable damage near the sensor–material interface before systematic quantitative testing could be completed. These observations do not allow the relative contributions of interfacial adhesion, bulk-material compliance, local stiffness differences, and fabrication conditions to be distinguished. Therefore, the Phase I configurations were not included in the subsequent quantitative comparison. Their main contribution was the identification of sensor integration and encapsulation as critical design considerations for subsequent configurations. This motivated the transition to rigid structural solutions with surface-mounted rather than embedded strain gauges.

### 3.2. Phase II—First Rigid Concept: Half-Shell

**Figure 6:**
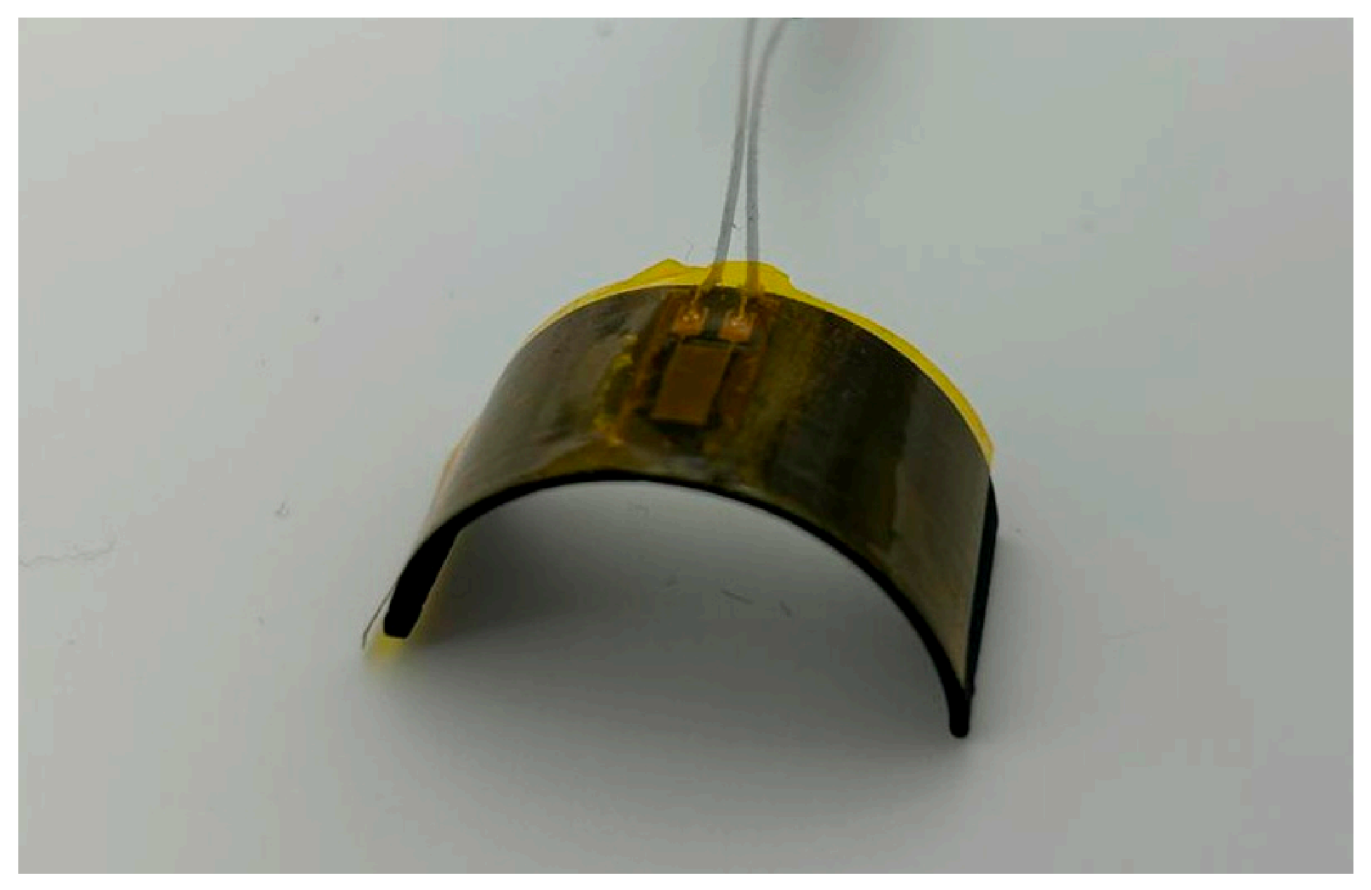
Half-shell geometry (real prototype).

The first rigid configuration consisted of a half-shell structure (Figure 6) manufactured in PETG using FFF technology. The geometry was designed as a clamp-type interface, allowing easy installation and removal without permanent attachment. The strain gauge (1-LY48-6/350V3, HBM, Darmstadt, Germany) was positioned along the shell’s longitudinal axis, aligned with the expected bending direction.

**Figure 7:**
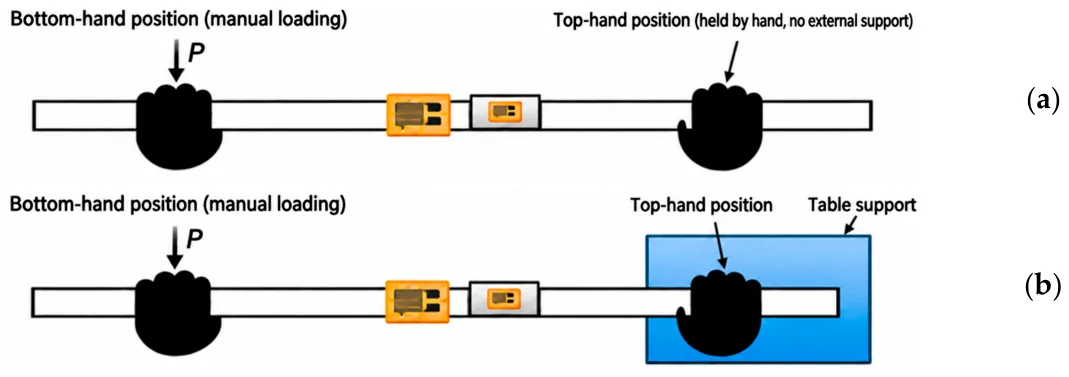

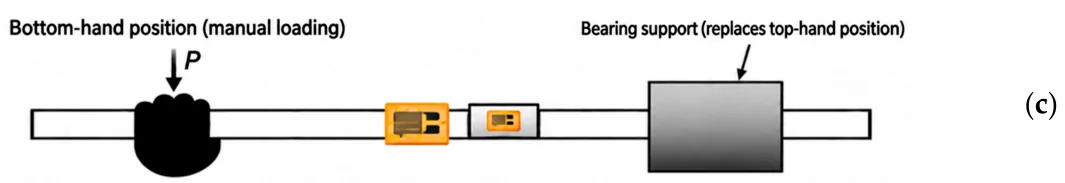
Exploratory setups used to evaluate the half-shell under three support conditions at the top-hand position: (**a**) hand-held, without external support; (**b**) table-supported; and (**c**) bearing-supported.

The transition from compliant to rigid configurations was motivated by the fabrication and sensor-integration limitations observed in Phase I, which prevented quantitative evaluation of the compliant concepts. The adoption of a rigid substrate enabled the strain gauge to be mounted on the interface surface rather than embedded within the material, thereby avoiding the encapsulation approach associated with the visually observed defects. A stiffer substrate was also expected to improve strain transmission, although this effect was not quantified in Phase I. To evaluate the mechanical behavior of the half-shell configuration, exploratory tests were conducted under three boundary conditions (Figure 7) differing in how the shaft was supported at the top-hand position while the load was applied manually at the bottom-hand position: (Figure 7a) both positions held by hand, without external support; (Figure 7b) the top-hand position resting on a table, introducing a contact constraint along the shaft; and (Figure 7c) the top-hand position replaced by a rigid bearing support. These tests were exploratory and used manually applied loading rather than calibrated weights. Of the three conditions, the bearing-supported configuration shown in Figure 7c provided the closest approximation to the rigid support subsequently used in the standard static configuration shown in Figure 2. However, the two procedures were not identical because loading in the Figure 7 tests remained manual. Because the metrics in this phase compare the shell response with the bonded reference gauge on the same shaft under identical loading, absolute force control was not required at this exploratory stage.

Correlation, gain (slope), offset, and coefficient of determination (R^2^) were used to assess the quality of strain transmission. The results are summarized in Table 3.

The results reveal a counterintuitive pattern: the table-supported condition, which adds a contact constraint along the shaft, produced the lowest R^2^ (0.48) despite providing greater overall structural support. Changes in the bending moment distribution along the shaft explain this behavior. Under the hand-held condition, the shaft deflects between two compliant supports, producing a deformation field that was nevertheless reproducible at the measurement location. Introducing a third contact point, the table surface, creates an additional reaction force whose position and magnitude are not precisely controlled, altering the bending moment diagram in a condition-dependent way that is difficult to reproduce between measurements. The bearing-supported configuration, while introducing localized support near the loading region, produced an intermediate R^2^ (0.51), consistent with a partial but imprecise modification of the load path. These results indicate that partial-contact geometries are highly sensitive to boundary conditions; any uncontrolled contact point alters the deformation field at the measurement location, compromising both accuracy and repeatability.

**Figure 8:**
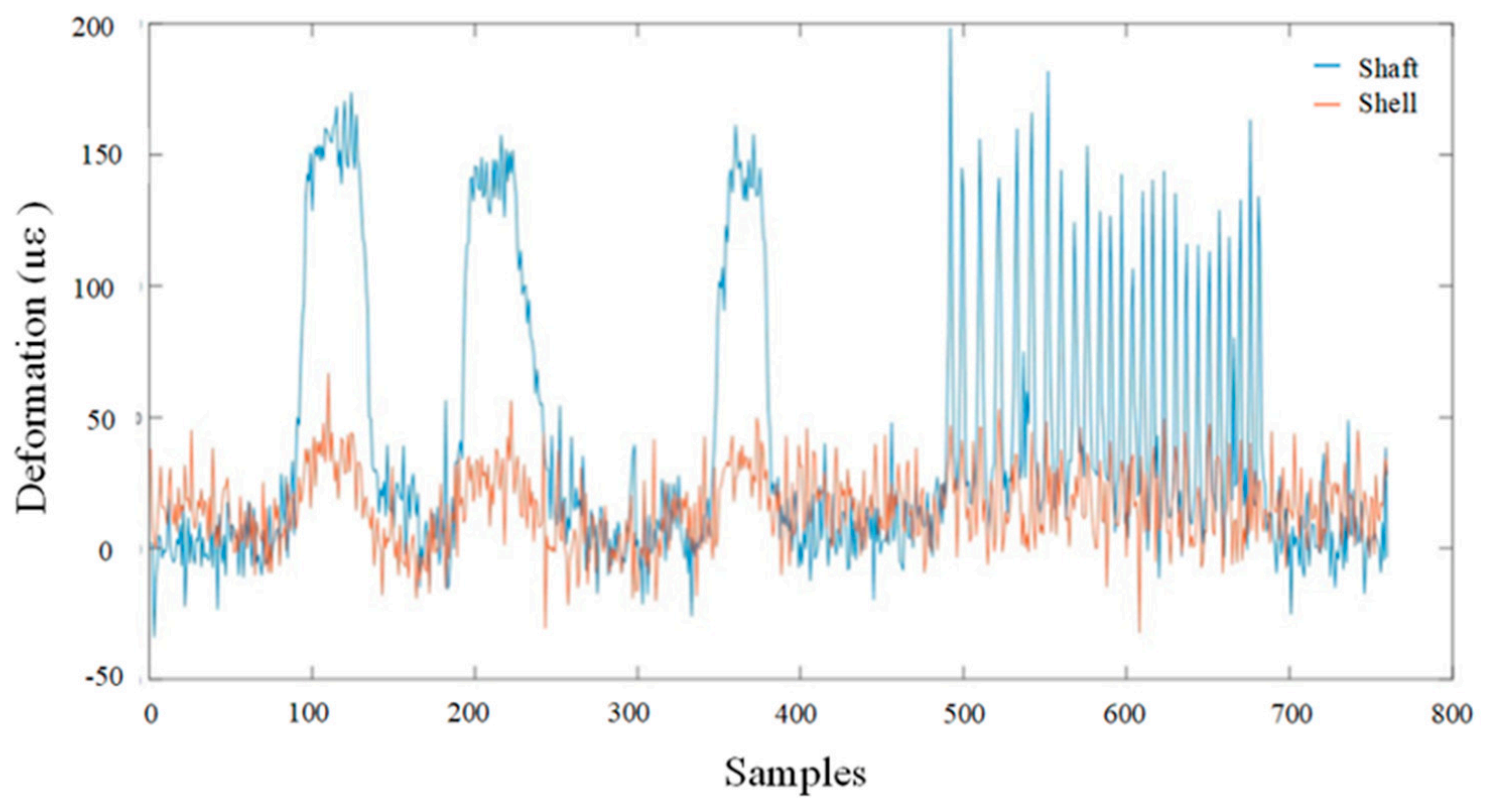
Dynamic strain response comparison between the bonded shaft reference (blue) and the half-shell configuration (orange), confirming near-complete signal attenuation under cyclic loading.

Dynamic testing further confirmed the limitations of the half-shell configuration. As shown in Figure 8, the reference signal (blue) exhibited well-defined force peaks with amplitudes reaching approximately 300 µε, clearly capturing the dynamic loading events. The half-shell signal (orange), however, remained heavily attenuated throughout the test, with a maximum amplitude of approximately 50–60 µε and no discernible reproduction of the reference peaks. The shell output was effectively buried in noise, indicating that partial circumferential contact is insufficient to transmit dynamic deformation events with any meaningful fidelity.

Taken together, the static and dynamic results confirm that partial circumferential contact leads to non-uniform load transfer, strong sensitivity to external constraints, and severe signal attenuation under dynamic loading. The maximum static R^2^ of 0.54 and the near-complete loss of dynamic signal fidelity establish that half-shell geometries are fundamentally inadequate for reliable strain measurement, motivating the development of fully enclosing geometries with continuous circumferential contact.

### 3.3. Phase III—Full Wrap-Around Shell

**Figure 9:**
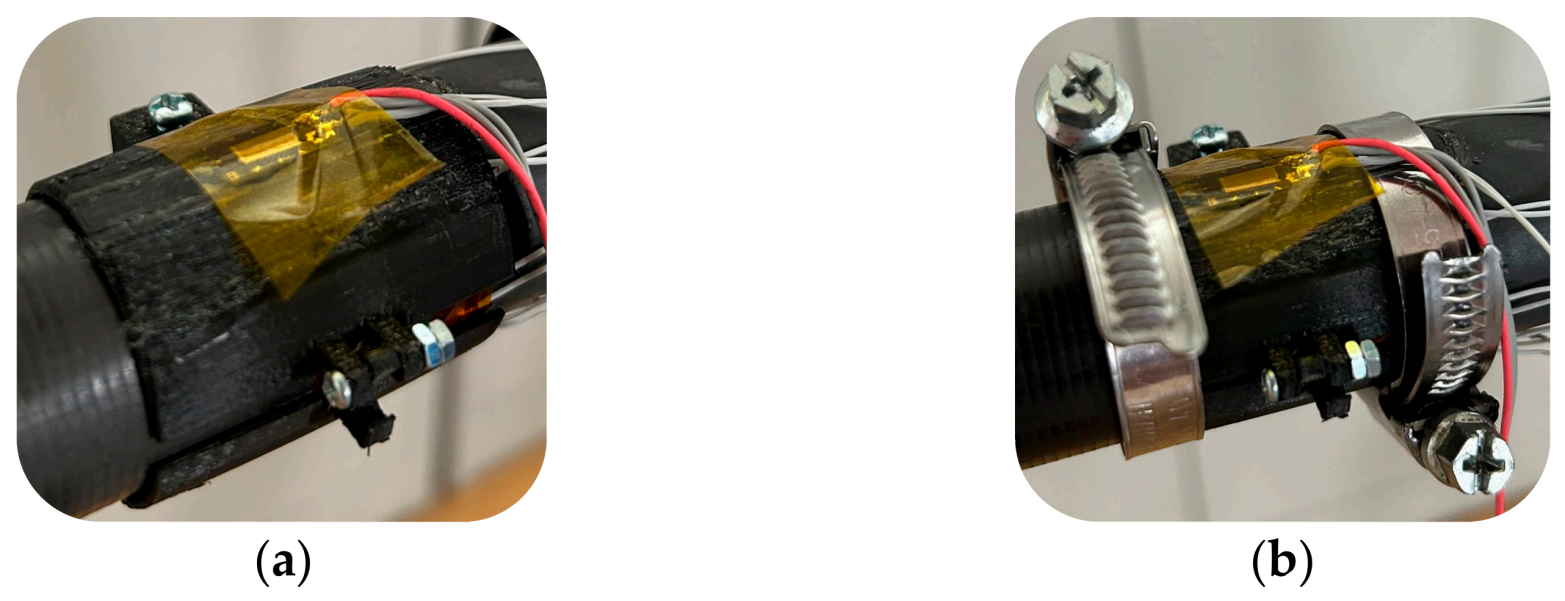
Full wrap-around shell geometry: (**a**) simple enclosing shell (iteration 4); (**b**) shell with additional external metallic clamp (iteration 5).

To overcome the limitations of partial contact, a full wraparound shell was developed to enclose the shaft and provide continuous circumferential contact. Two variants of this configuration were evaluated simultaneously (Figure 9): a simple enclosing shell (Figure 9a) and a shell with an additional external metallic clamp (Figure 9b).

Experimental characterization was carried out under controlled laboratory conditions, using the static setup described in Section 2 (standard static configuration, Figure 2) over the 0–300 N range. Each load step was held for 30 s to achieve steady-state readings. Raw ADC output from both shell variants and the bonded reference gauge was recorded synchronously using the same acquisition chain; no conversion to physical units was applied, enabling a direct comparison between the two measurement systems under identical conditions. The sensitivity of the Phase III configurations—and of all subsequent configurations—to alternative shaft support conditions was not evaluated (see Section 5).

Agreement between signals was evaluated using the coefficient of determination (R^2^) and hysteresis, as summarized in Table 4. The bonded reference achieved an R^2^ of 0.99 and a hysteresis of 3.41 µε, establishing the performance baseline for comparison.

**Figure 10:**
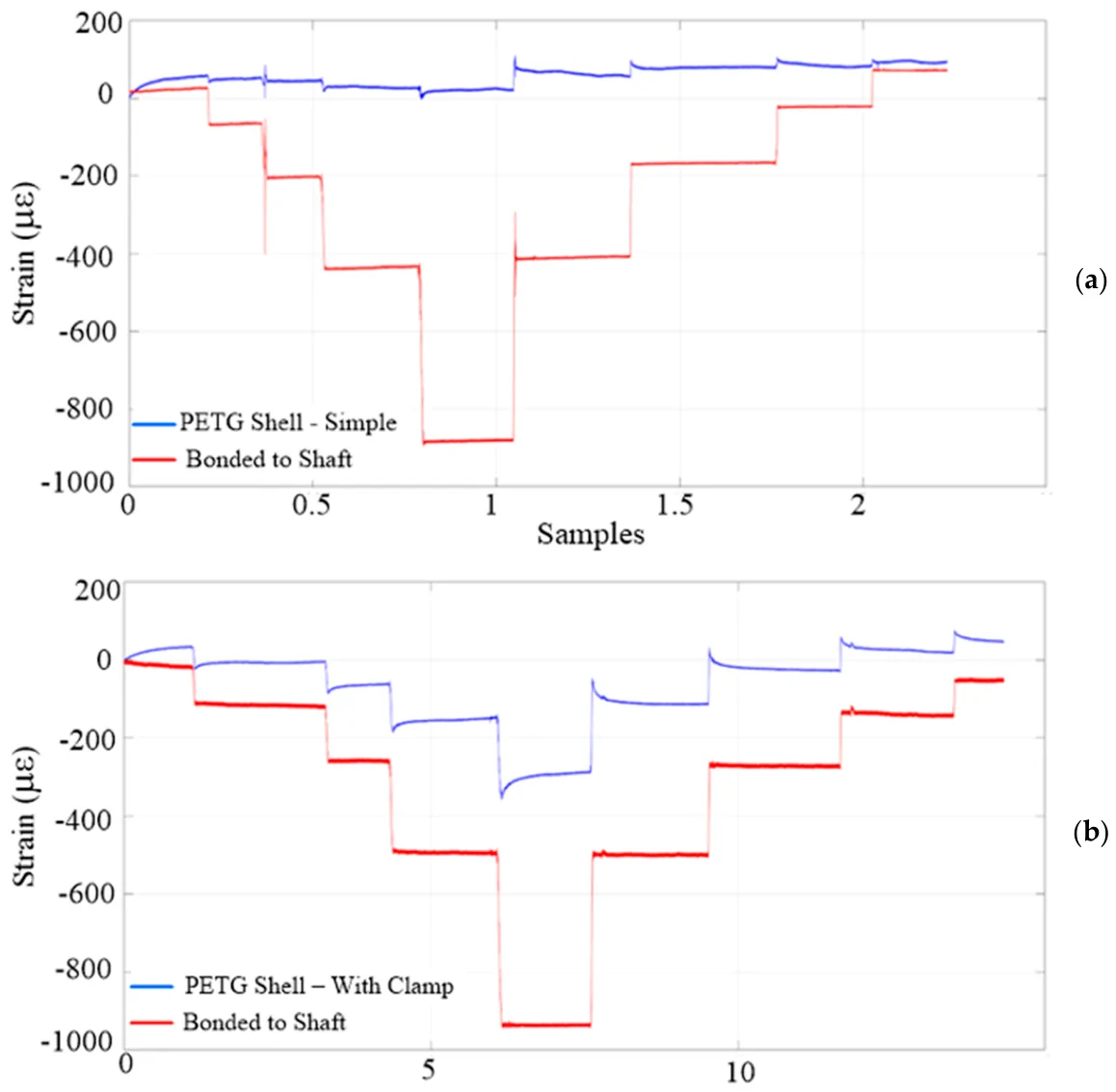
Static strain comparison between shaft and full wrap-around shell under multi-level incremental loading: (**a**) simple shell; (**b**) shell with clamp.

As shown in Figure 10a, the simple shell failed to track the reference signal: the shell output (blue) remained nearly constant. At the same time, the bonded gauge (red) responded clearly to each incremental load step, resulting in poor linearity (R^2^ = 0.41). This behavior confirms that circumferential enclosure alone is insufficient to ensure reliable strain transmission. Without an active clamping mechanism, contact pressure around the shell remains non-uniform, promoting localized micro-slip under cyclic loading [20,21], which effectively decouples the shell from the shaft deformation.

The addition of a metallic clamp produced a substantial improvement, with the shell signal now closely following the stepwise loading pattern of the reference gauge (R^2^ = 0.97, Figure 10b). However, hysteresis remained elevated at 34.21 µε, approximately ten times above the bonded reference value of 3.41 µε, suggesting that a single clamping point, while effective in improving contact stiffness, generates a non-uniform circumferential pressure distribution that continues to promote energy dissipation through micro-slip.

These results establish two key conclusions: full circumferential enclosure is necessary but not sufficient for acceptable strain transmission, while a single clamping point is insufficient to suppress hysteresis to levels compatible with accurate measurement. Together, these findings indicate the need for a distributed clamping strategy, motivating the next design iteration: a full-length continuous hinge combined with two separate closure points to distribute clamping forces more uniformly and reduce the residual micro-slip identified in this phase.

### 3.4. Phase IV—Structural Optimization and Clamping Strategy

**Figure 11:**
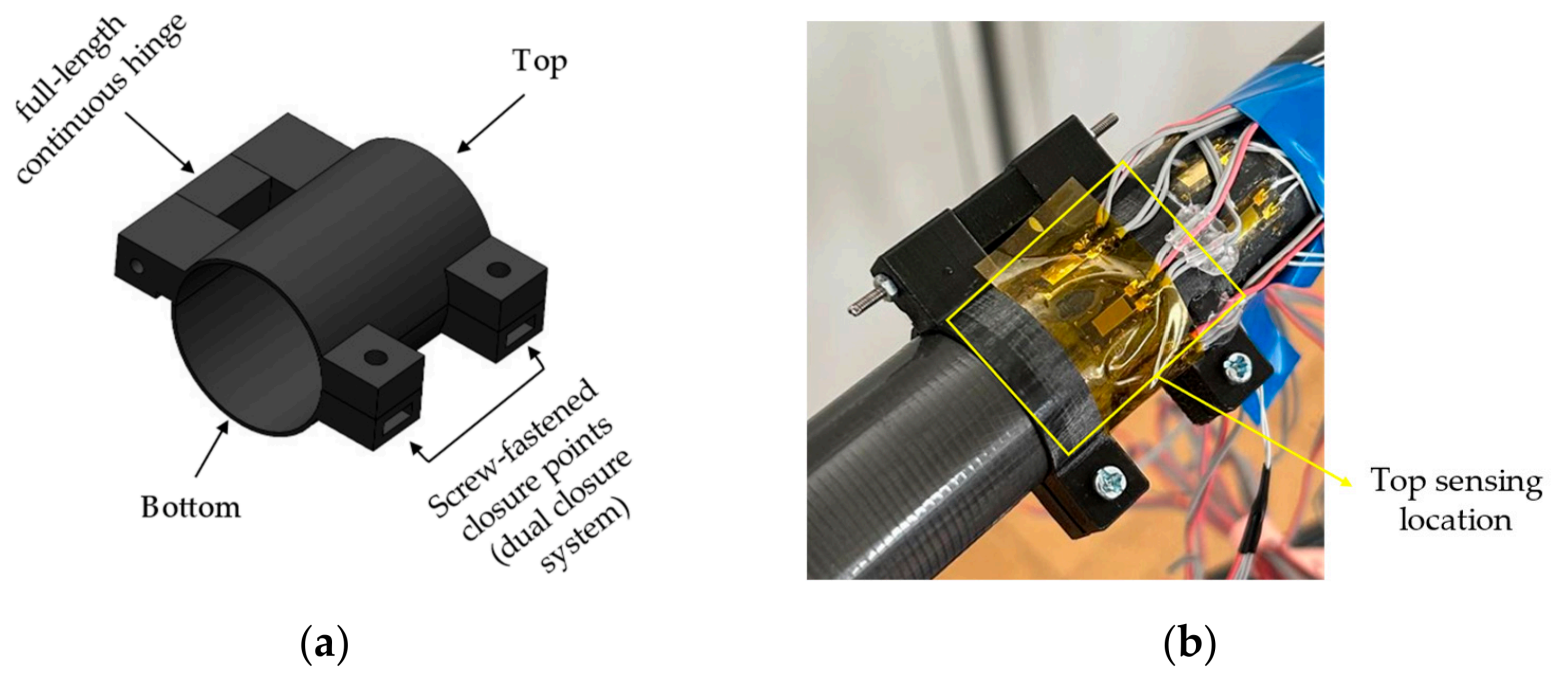
Shell design with full-length continuous hinge and dual closure system (iteration 6): (**a**) 3D CAD model, showing the full-length continuous hinge and the two screw-fastened closure points; (**b**) real prototype mounted on the shaft, showing the top sensing location.

To eliminate the residual hysteresis observed earlier, the clamping approach was thoroughly overhauled. A new design featuring a full-length continuous hinge on the shell and two separate screw-fastened closure points (hereafter, the dual closure system) was created (see Figure 11). The hinge ensured smooth, repeatable shell closing, preventing cross-sectional deformation and minimizing misalignment between the shell and shaft during installation. The two closure points distribute clamping forces along the length, resulting in more uniform circumferential contact pressure and reducing the local pressure peaks typically caused by single-point clamping.

**Figure 12:**
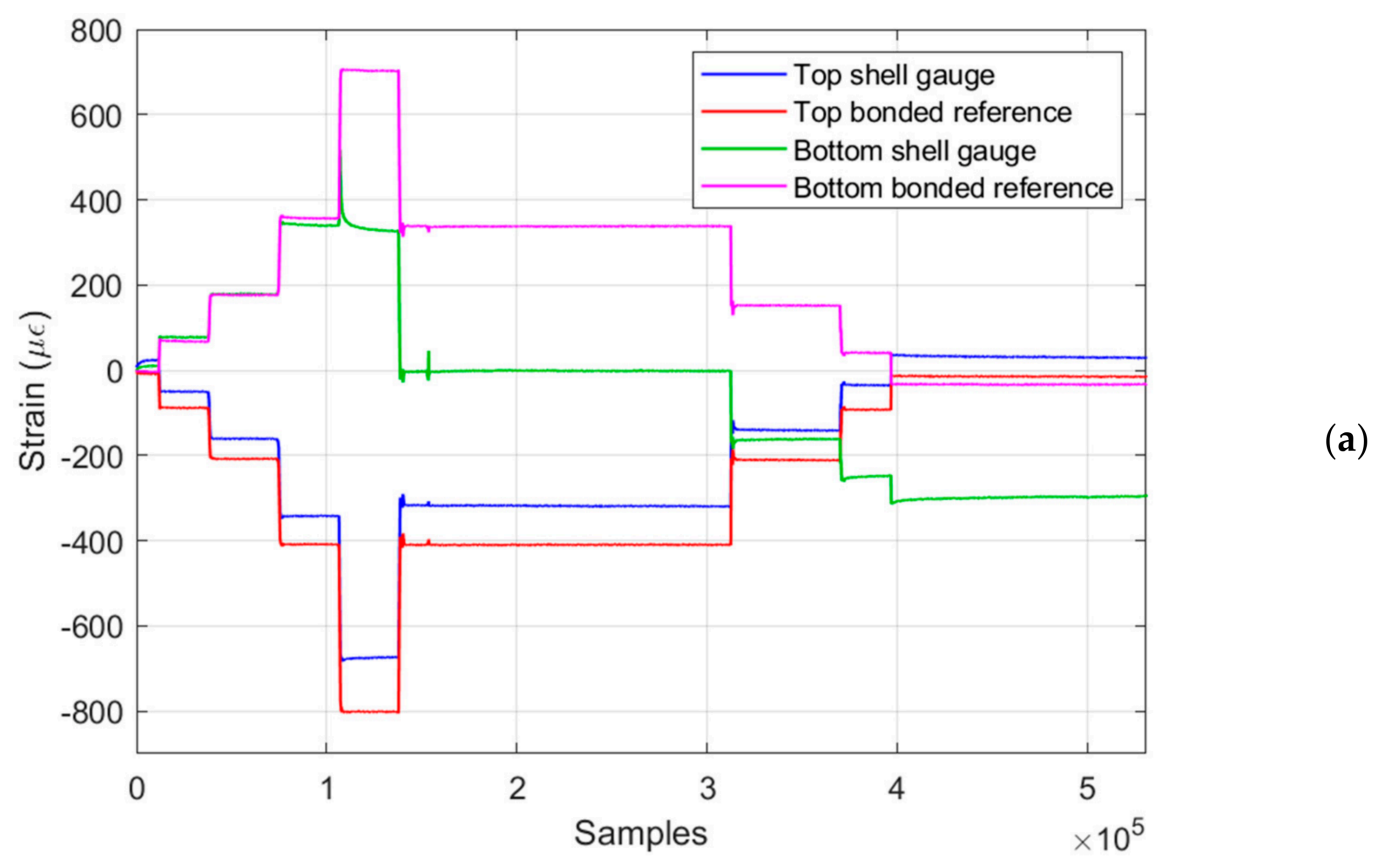

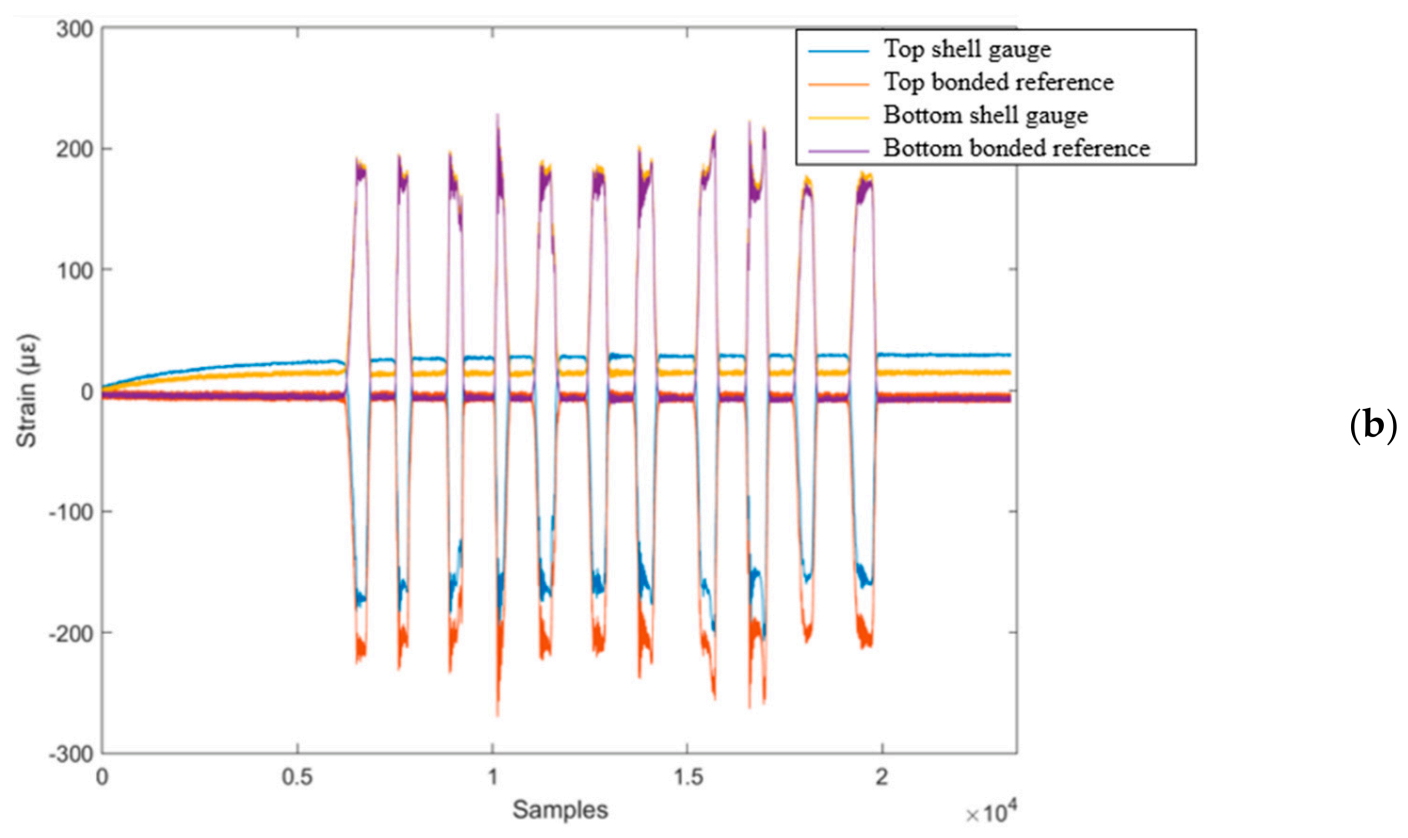
Strain response of the hinge-and-dual-closure shell (iteration 6): comparison between the top and bottom shell gauges and the corresponding top and bottom bonded reference gauges on the shaft under (**a**) incremental static loading (0–200 N) and (**b**) cyclic loading. The bonded reference gauges represent the bending response of the shaft at the two diametrically opposed locations; differences between the top and bottom shell signals reflect interface-driven effects (local shell–shaft contact and clamping non-uniformity) rather than shaft asymmetry.

Static tests were performed following a protocol similar to that described in Section 3.3, with the load range reduced to 0–200 N and strain responses recorded synchronously from both the shell and the bonded reference gauge. Static characterization revealed a marked dependence of measurement performance on sensing location (Figure 12a and Table 5). R^2^ was calculated between the applied load and the mean strain response at each loading and unloading plateau. Hysteresis was calculated as the mean absolute difference between the loading and unloading responses at corresponding load levels, excluding the maximum load. MAE and RMSE represent residual force errors obtained after fitting a linear force–strain relationship to the plateau values.

Throughout this section, “top” and “bottom” denote the two diametrically opposed sensing platforms integrated into the shell, offset by 180° around the shaft circumference and aligned with the bending plane of the static test. The “top” location corresponds to the upward-facing platform in the mounted configuration shown in Figure 11b, and the “bottom” location corresponds to the opposing platform on the underside of the shell. Both sensing locations are positioned at the same axial coordinate along the shaft so that the differences reported below reflect circumferential rather than axial position.

The top shell gauge closely followed the bonded reference, presenting R^2^ = 0.998, MAE = 2.26 N, RMSE = 2.47 N, and hysteresis of 16.80 µε. In contrast, the bottom shell gauge presented R^2^ = 0.484, MAE = 37.23 N, RMSE = 43.75 N, and hysteresis of 329.75 µε, indicating substantial deformation accumulation during unloading. Thus, although the dual-closure strategy provided a stable response at one sensing location, it did not produce uniform strain transmission across the shell. The bonded reference gauges on the shaft presented a highly linear response at both diametrically opposed locations (R^2^ = 1.000 and 0.997; Table 5), consistent with the expected symmetrical bending response of the shaft. Although the bottom bonded reference also showed higher hysteresis than the top bonded reference (25.24 vs. 3.54 µε), this difference was approximately one order of magnitude smaller than the hysteresis of the bottom shell gauge (329.75 µε). Therefore, the discrepancy between the top and bottom shell signals, particularly the pronounced hysteresis observed at the bottom location, cannot be attributed to an asymmetrical mechanical response of the shaft. Instead, it reflects interface-driven effects associated with local shell–shaft contact conditions, non-uniform clamping pressure, and micro-slip during unloading. The shell was subsequently tested under dynamic simulation conditions using the same mechanical setup, with the load applied at the bottom-hand position to reproduce cyclic loading representative of a paddle stroke (Figure 12b). Visual comparison showed that the shell signals followed the main timing of the repeated loading cycles, while differences in amplitude, baseline, and cycle-to-cycle response remained evident.

The observed hysteresis accumulation, signal drift, and differences between the top and bottom sensing locations were therefore attributed to local shell–shaft contact conditions and clamping non-uniformity, rather than to asymmetry of the shaft response.

**Figure 13:**
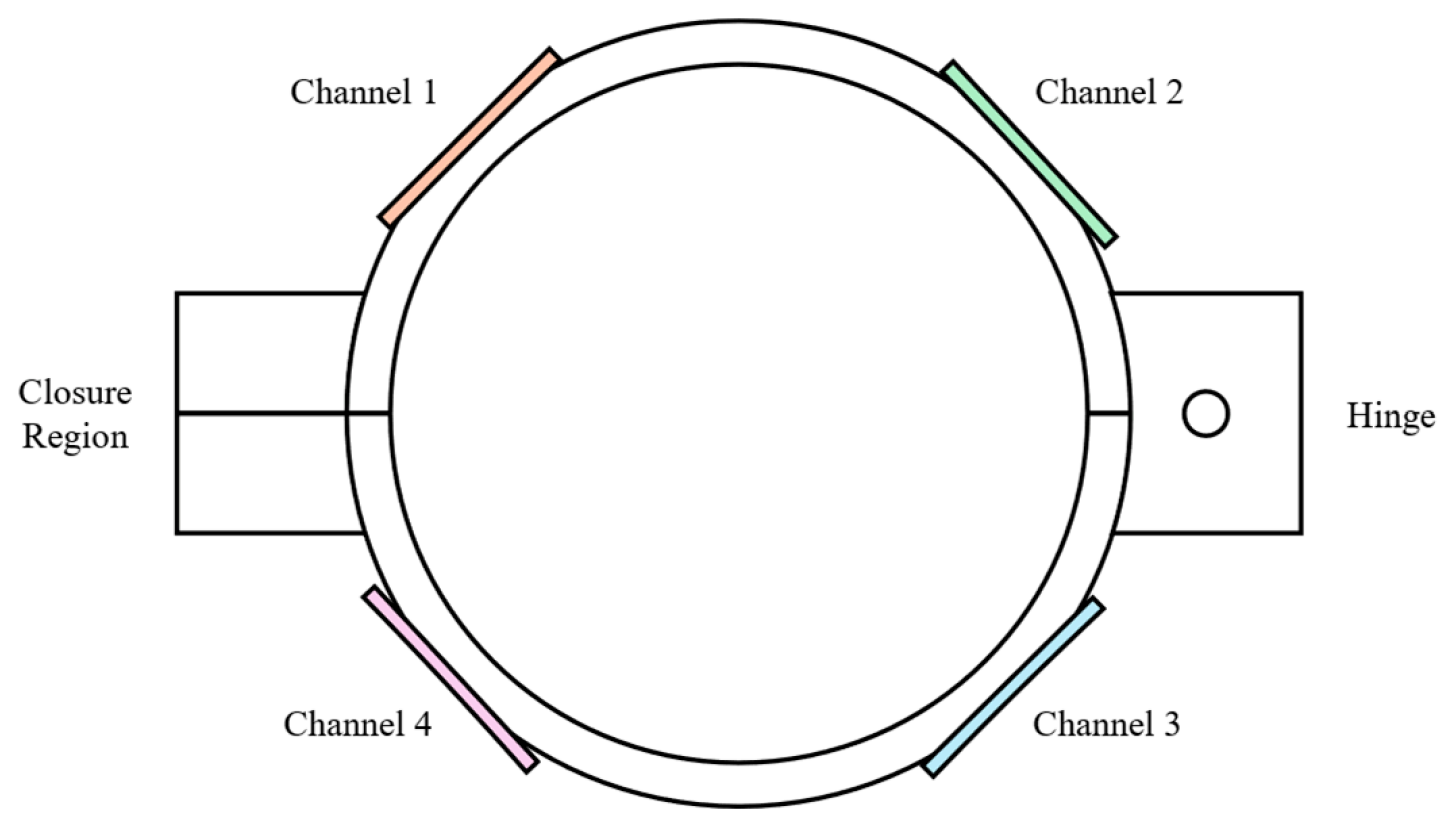
Schematic visualization of the shell: two strain gauges in the screw-closure region (screw-side channels 1 and 4) and two near the hinge (hinge-side channels 2 and 3).

The optimized shell was subsequently evaluated under on-water field conditions. One female athlete with extensive competitive experience in sprint kayaking participated in the evaluation. The use of a single participant represents a limitation of this phase. However, the main objective was to verify the capacity of the developed system under real training/competition conditions. Therefore, the on-water results should be interpreted as indicative of system behavior under field conditions rather than as a characterization of performance across different athletes or paddling styles. Moreover, because no independent force reference was available and the field test involved a single athlete, the reported metrics reflect internal signal consistency rather than absolute on-water force accuracy. Tests were performed in a K1 kayak at a self-selected paddling intensity over a 50 m course. Four strain gauges were installed symmetrically on the shell: two in the screw-closure region (screw-side channels 1 and 4) and two near the hinge (hinge-side channels 2 and 3), enabling direct comparison between zones with different local contact conditions and structural stiffness (Figure 13). The study was conducted in accordance with the Declaration of Helsinki and was approved by the Ethics Committee of the Faculty of Sport Sciences and Physical Education, University of Coimbra (FCDEF-UC) (approval no. CE/FCDEF-UC/00062024); the participant provided written informed consent before data collection.

**Figure 14:**
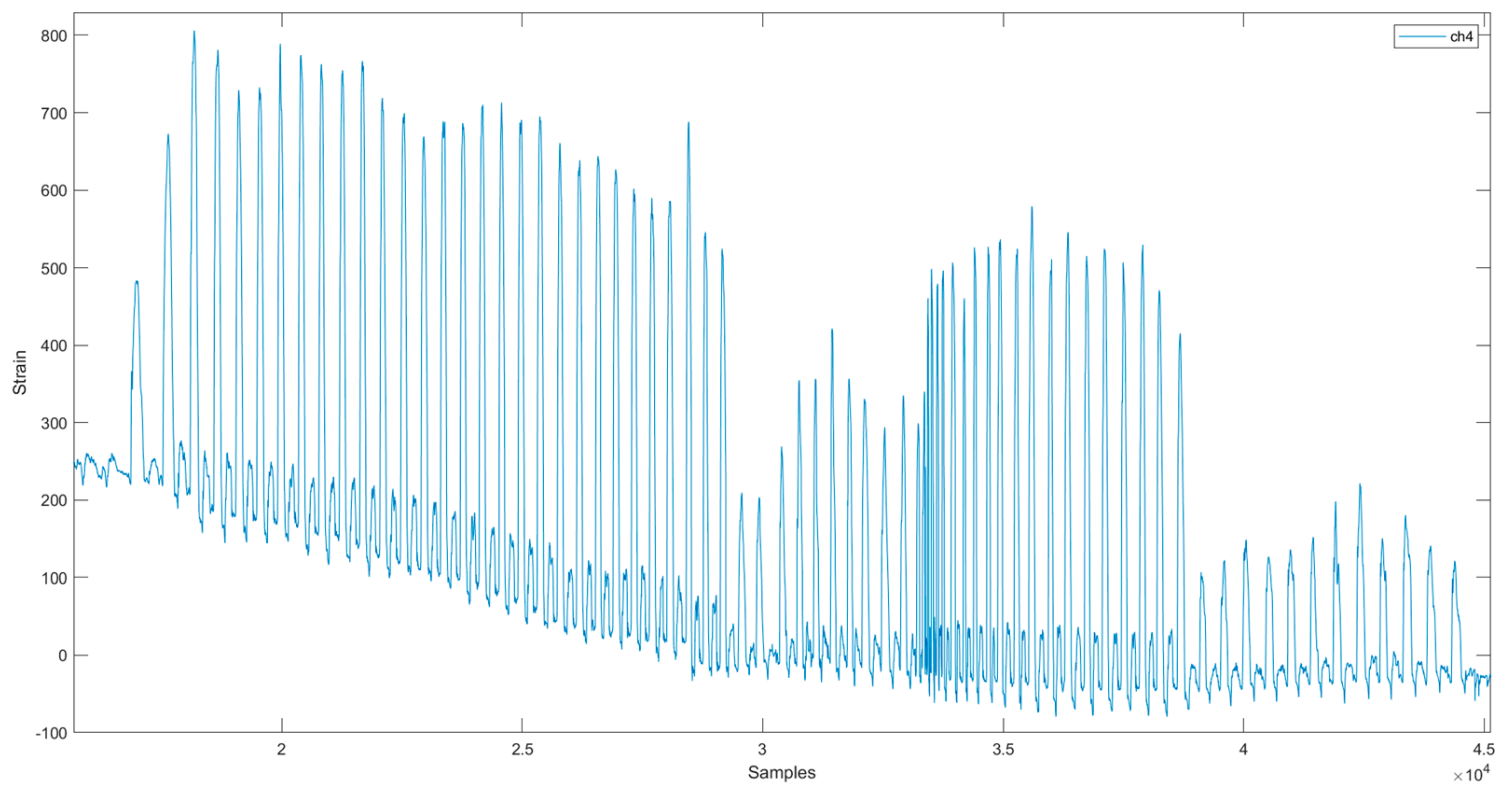
On-water strain signals from a single test repetition, illustrating the cyclic pattern corresponding to the paddling stroke and the variation in signal amplitude with stroke intensity.

The acquired signals initially exhibited instability during the early phase of data acquisition, attributed to mechanical settling of the interface in the absence of a defined reference state. Following a zeroing procedure, the signals stabilized, and well-defined cyclic patterns corresponding to the paddling stroke were clearly identifiable. Variations in signal amplitude reflected changes in stroke intensity, confirming that the interface retained sensitivity to biomechanically relevant variations in force (Figure 14).

**Figure A1:**
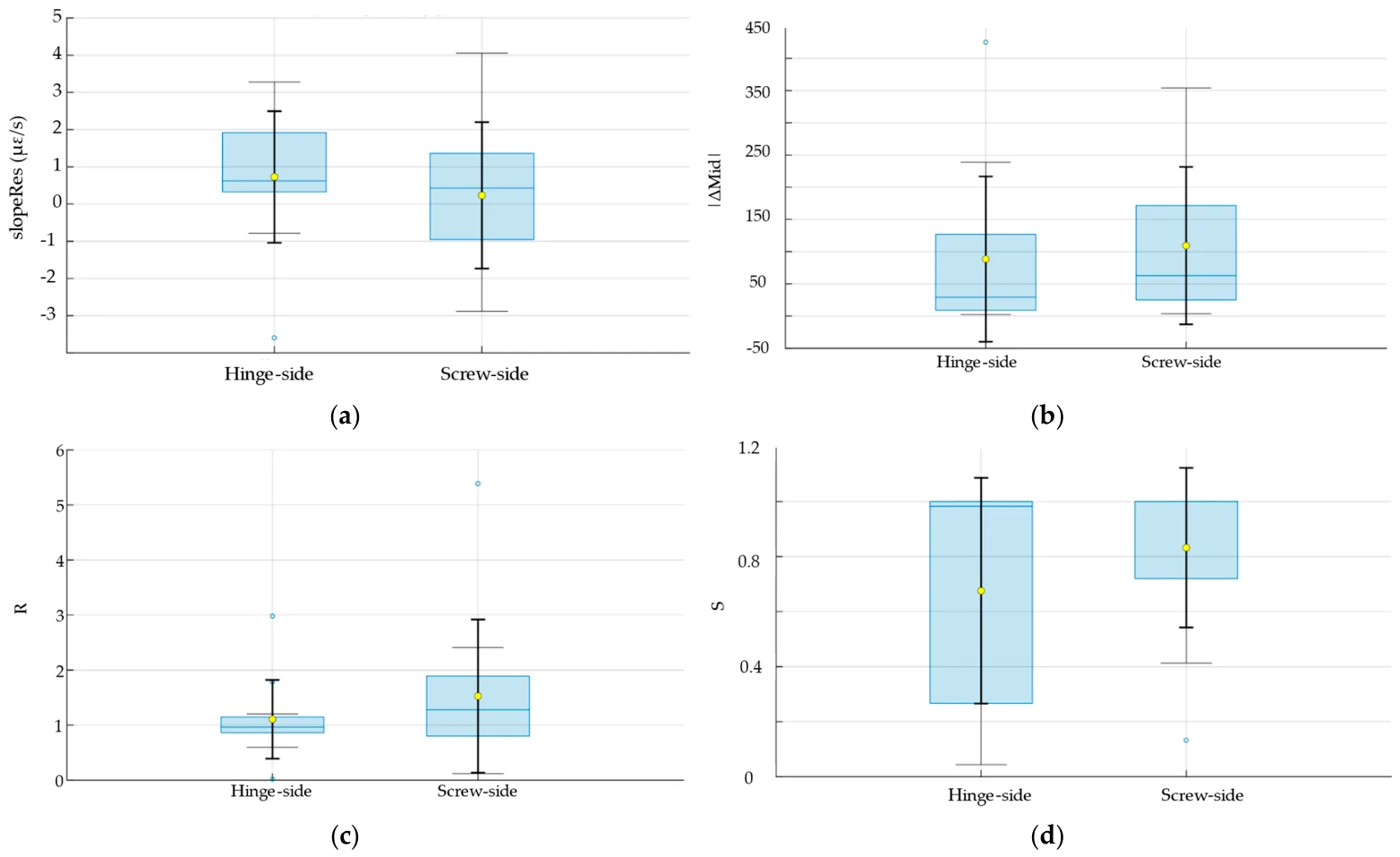
Distributions of on-water measurement quality metrics grouped by closure type (hinge-side vs. screw-side channels): (**a**) residual slope (slopeRes, µε/s); (**b**) absolute mid-point offset (|ΔMid|, µε); (**c**) proportionality ratio (R); (**d**) symmetry index (S). Boxes show the median and interquartile range; whiskers extend to 1.5×IQR.

To quantitatively characterize the influence of sensor position on measurement quality, the four complementary on-water metrics defined in Section 2 were computed for each channel across all test repetitions, and their distributions were grouped by closure type (Figure A1). The metrics revealed two distinct and complementary uncertainty signatures, one associated with each closure mechanism: hinge-side channels exhibited systematically positive residual slopes (slopeRes), progressive hysteresis accumulation across stroke cycles, but a consistent gain relationship with the reference, whereas screw-side channels showed centered residual slopes but a proportionality ratio (R) spanning a factor of ≈3.6 across installations and larger absolute mid-point offsets (|ΔMid|), reflecting the sensitivity of the screw contact state to mounting torque and positioning. These signatures are mechanically consistent with the contact framework established throughout this work: the hinge introduces a localized geometric discontinuity with finite clearance, superimposing fretting-type drift on the adjacent gauges, whereas the screw closure produces an installation-dependent contact state. Neither mechanism eliminates measurement artifacts; each introduces a different type of uncertainty.

Taken together, these on-water results indicate that the shell-based configuration with distributed clamping can capture the temporal structure of the paddling stroke and respond to variations in applied force under realistic operating conditions, within the constraints of a single-participant evaluation. These observations motivated a shift in design philosophy: rather than continuing to optimize the clamping of a fully enclosing shell, the following phase explored simplified structural geometries in which the load path and boundary conditions could be more precisely controlled, reducing the dependence of measurement quality on closure-related contact phenomena.

### 3.5. Phase V—Beam-Based and Hybrid Configurations

The limitations identified in Phase IV, particularly the dependence of measurement quality on closure-related contact phenomena and the inability of a fully enclosing shell to suppress hysteresis solely through clamping optimization, motivated a fundamental shift in design philosophy. Rather than relying on distributed circumferential contact, this phase explored beam-based elements mounted on the shaft, thereby concentrating deformation within a defined structural pathway and enabling more precise control of boundary conditions.

**Figure 15:**
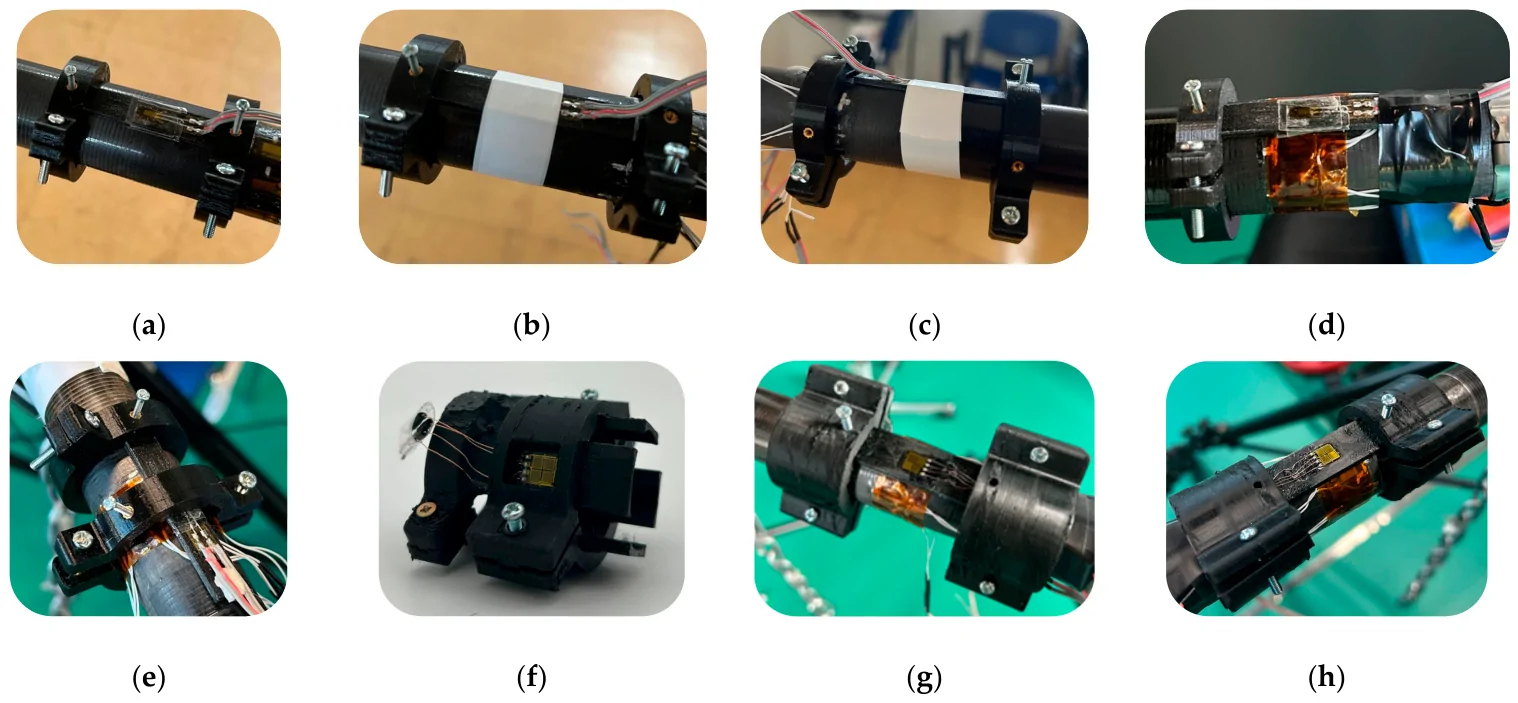
Overview of beam-based interface configurations (iterations 7–14): (**a**) it. 7—doubly fixed by two rings; (**b**) it. 8—with adhesive tape between shaft and rings; (**c**) it. 9—one ring fixed, free end, with tape; (**d**) it. 10—free end variant; (**e**) it. 11—ring repositioned over sensor; (**f**) it. 12—ring with open window over sensor; (**g**) it. 13—increased beam and ring width; (**h**) it. 14—increased width with double-sided tape.

A total of eight configurations were developed and evaluated in this phase (iterations 7–14), all based on curved beam elements conforming to the shaft geometry. Static tests were performed following a protocol similar to that described in Section 3.3, with the shaft loaded incrementally from 0 to 300 N and strain responses recorded synchronously from both the beam-based interface and the bonded reference gauge. Performance was assessed using four metrics: linearity (R^2^), hysteresis (raw ADC units), coefficient of variation of the mean signal (CV, %), and full-scale output error (FSO, %). Note that hysteresis values in this phase are reported in raw ADC units and are not directly comparable to the µε values reported in Phases III and IV. To contextualize the relative magnitude of hysteresis within this phase, the full-scale output error (FSO, %) also reported in Table 6 provides a measure of the total signal range for each configuration; hysteresis values should therefore be interpreted relative to the corresponding FSO rather than as absolute quantities. The configurations explored in iterations 7–14 are illustrated in Figure 15.

As shown in Figure 15, the first group (iterations 7–12) maintained the same curved-beam geometry while systematically varying the fixation strategy, whereas the second group (iterations 13–14) also increased the beam and ring widths. The quantitative results for all configurations are summarized in Table 6.

As shown in Table 6, no fixation strategy resolved the trade-off between linearity and hysteresis. The doubly fixed configurations achieved the highest linearity of the group (R^2^ = 0.879–0.904, iterations 7, 13, and 14), but retained elevated hysteresis, whereas partially decoupled variants (iterations 9–11) reduced hysteresis by up to a factor of four at the cost of unacceptable linearity (R^2^ = 0.440–0.586). Interposing compliant layers (double-sided tape, iterations 8 and 14) or repositioning the rings relative to the sensor (iterations 11–12) shifted the balance between the two metrics without ever satisfying both: configurations that stabilized the load path introduced constraint-induced distortion at the ring–beam interface; configurations that relieved that constraint lost stiffness in the load path.

**Figure 16:**
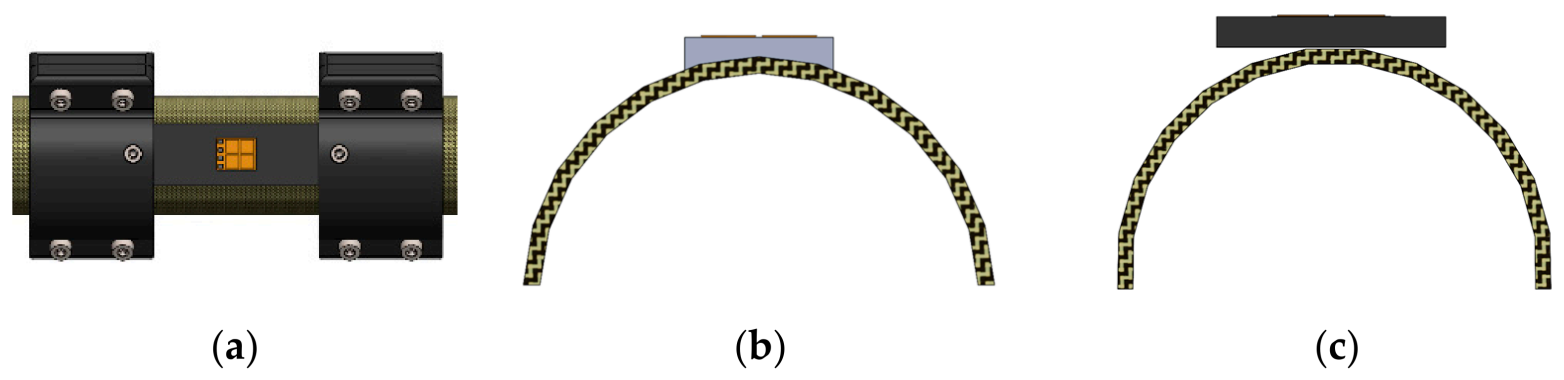
Shell-integrated beam configurations (iterations 15–16): (**a**) shell prototype mounted on the shaft, with external geometry common to both variants; (**b**) curved beam cross-section (iteration 15); (**c**) straight beam cross-section (iteration 16).

The critical insight from this phase was that the limitation was not the beam geometry itself but the boundary conditions imposed by the rings. Discrete fixation creates localized constraint zones that distort the deformation field at the sensor location, and no arrangement of ring position, width, or adhesive layer could resolve this. The logical response was therefore not to continue optimizing ring-based fixation, but to eliminate the rings by integrating the beam element directly within a shell structure. This approach combined the geometric stability of a full enclosure, which distributes contact forces more uniformly along the shaft, with the controlled deformation pathway of a beam, replacing discrete constraint points with a continuous structural boundary condition. Iterations 15 and 16, illustrated in Figure 16a, implemented this concept in two beam cross-sections—curved (Figure 16b) and straight (Figure 16c)—to isolate the effect of beam geometry independently of fixation strategy.

For these configurations, the evaluation protocol was refined to include an independent S-type load cell (TS 50 kg, OIML C2; AEP transducers, Modena, Italy) as the force reference. The bonded strain gauge used in the preceding phases was appropriate for comparing how closely the removable interfaces reproduced the local shaft-deformation response, whereas the load cell enabled direct quantification of force-magnitude errors in the shell-integrated beam configurations. This change does not affect the ranking within each development stage, since the configurations compared at each stage were evaluated using the same reference and protocol; however, quantitative results obtained using the two reference systems were not directly compared. This allowed direct quantification of force-magnitude errors, in addition to the relative signal-fidelity metrics used in previous phases. Static tests were conducted over a load range of 0–300 N using calibrated weights, verified by the load cell, as ground truth, and dynamic tests consisted of continuous cyclic loading applied simultaneously to all measurement systems. The static performance metrics for both configurations are summarized in Table 7.

The PETG curved beam configuration (iteration 15) eliminated the discrete ring constraints identified as a structural limitation in the preceding designs. Under the load-cell-based evaluation protocol, this configuration presented an MAE of 17.02 ± 0.63 N and an RMSE of 18.66 ± 0.54. Repeatability was moderate, with a CV of 3.7 ± 2.6% and hysteresis of 13.70 ± 1.30% of the applied load range, reflecting the sensitivity of the curved beam geometry to contact distribution variability between test sessions, consistent with the positioning sensitivity identified in earlier iterations of this phase.

The largest errors in both configurations occurred at zero load, particularly during unloading, when residual deformation was not fully recovered. This behavior was also present in the bonded reference. Still, it was substantially amplified in the shell configurations (Table 7), indicating that the zero-load error has two components, namely an intrinsic shaft component present in all configurations, and an interface-dependent component arising from micro-slip at low contact forces, which is specific to the removable shell and dominant at low load levels.

The PETG straight beam configuration (iteration 16) consistently demonstrated superior static performance, with a lower MAE (13.05 ± 0.09 N), RMSE (14.52 ± 0.09 N), CV (0.5 ± 0.8%), and hysteresis (9.8 ± 1.7% vs. 13.7 ± 1.3% of the applied load range) relative to the curved beam. The straight beam cross-section provides a more uniform bending stiffness distribution across the sensing region, reducing sensitivity to variability in contact positioning and resulting in more consistent force transmission between test sessions. Both configurations showed substantially higher MAE and RMSE than the bonded reference (MAE = 0.74 ± 0.39 N, RMSE = 0.79 ± 0.41 N), confirming that cross-session calibration error remains the dominant source of measurement uncertainty in beam-based configurations.

**Figure 17:**
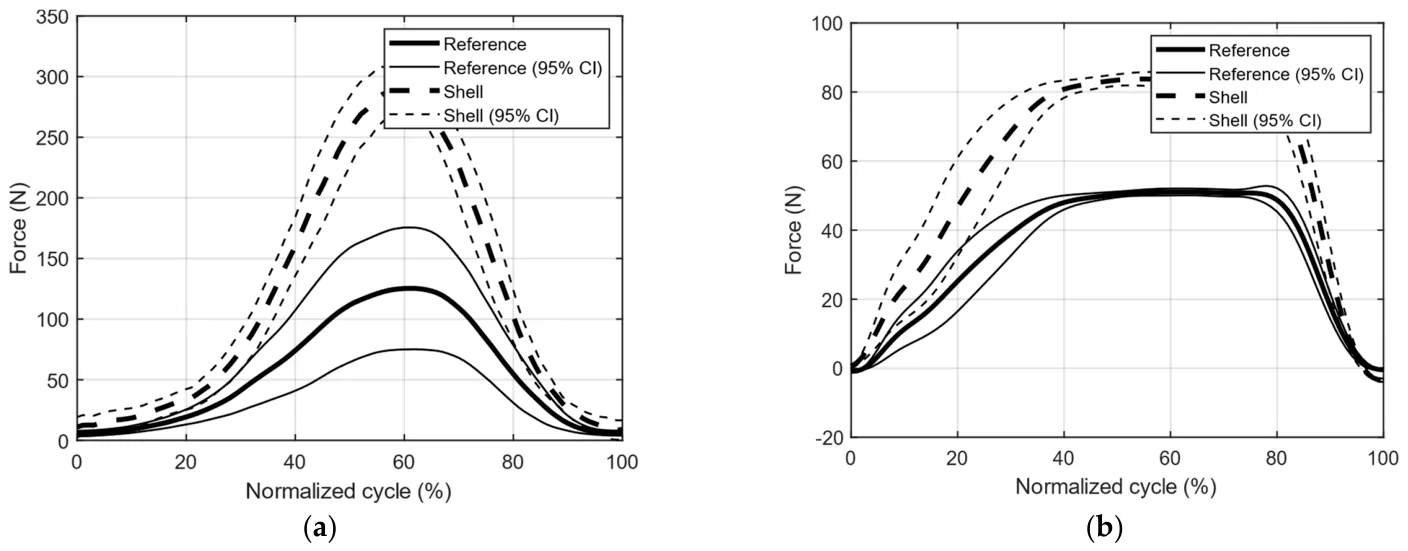
Mean curve graphs coupled with confidence intervals for (**a**) PETG—curved beam, (**b**) PETG—straight beam. The thick lines represent ±95% CI of each shell.

Dynamic evaluation further confirmed the superiority of the straight beam geometry. As shown in Figure 17a, the PETG curved beam exhibited wider confidence intervals throughout the cycle, particularly in the peak-force region, reflecting high cycle-to-cycle variability. The PETG straight beam showed the narrowest confidence intervals throughout the cycle, with a mean force curve closely following the reference sensor in temporal shape, with differences primarily concentrated at peak magnitude rather than in the signal’s temporal evolution (Figure 17b).

**Figure 18:**
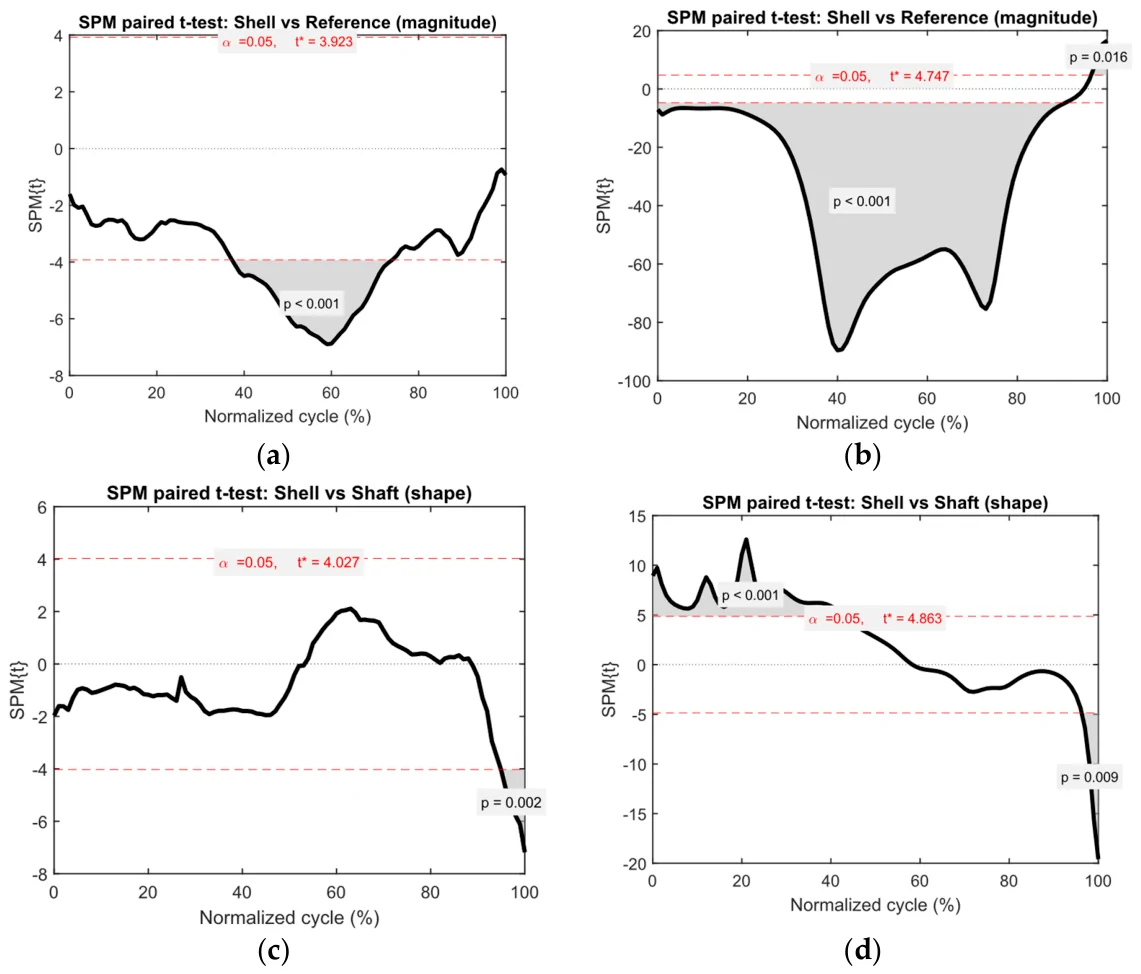
SPM analysis throughout the stroke cycle, comparing shell measurements against the load cell reference: force magnitude for (**a**) the PETG curved beam and (**b**) the PETG straight beam; time-normalized shape for (**c**) the curved and (**d**) the straight beam. Gray areas indicate regions of statistically significant differences between the shell and the reference.

Statistical Parametric Mapping (SPM) analysis, performed in MATLAB using the open-source spm1d package confirmed these observations [22]. For the PETG curved beam (Figure 18a), statistically significant differences in force magnitude were concentrated around the peak force region (*p* < 0.05). For the PETG straight beam, the significant differences were more localized, primarily in the maximum force region, while the remaining portions of the cycle showed no significant differences (Figure 18b).

Shape analysis further revealed that the PETG straight beam showed no statistically significant differences across most of the cycle (Figure 18c), indicating strong preservation of the temporal pattern of force application, the only configuration among those tested to achieve this result.

To further assess calibration stability across repeated test sessions, the calibration equation obtained from the first reference trial for each configuration was applied to the remaining trials for the same shell. This cross-session characterization allowed the inter-trial variability of the interface response to be quantified independently of fitting error. Table 8 summarizes the results.

When evaluated with its own session calibration, the bonded reference reproduced the applied force with a mean error of approximately 1%, establishing the error floor of the measurement chain. However, when the reference-session calibration was applied to independent test trials of the shells, mean errors rose to approximately 14% for the straight beam and 20% for the curved beam (Table 8). These results indicate that, although the straight beam improved calibration stability relative to the curved beam, the calibration coefficients were not fully transferable across sessions. Therefore, session-specific calibration remains necessary when accurate absolute force measurements are required. The suitability of these error levels for training monitoring depends on the intended application and on the required measurement accuracy, which were not defined as acceptance thresholds in the present study. In practical terms, errors of this magnitude are acceptable for monitoring of relative trends or load progression, but they constrain applications that require accurate absolute force quantification unless session-specific recalibration is performed.

This contrast is mechanically consistent with the contact behavior characterized throughout this study, where the straight beam concentrates deformation within a defined structural element, providing a more repeatable load path between sessions. In contrast, the curved beam is more sensitive to the precise contact distribution between shell and shaft, inheriting the positioning sensitivity identified in earlier phases. Elevated errors at low loads and during unloading in both configurations are consistent with micro-slip at the interface.

The results of iterations 15 and 16 established that a straight beam cross-section provided superior static performance relative to curved geometries, with lower absolute error, lower hysteresis (9.8 ± 1.7% vs. 13.7 ± 1.3% of the applied load range), and strong repeatability (CV 0.5 ± 0.8%). However, SPM analysis revealed statistically significant differences in force magnitude during the peak-loading portion of the paddle stroke’s propulsive phase, where biomechanically relevant force information is concentrated. A configuration that performs well across most of the cycle but introduces systematic error at peak force does not satisfy the core instrumentation requirement, regardless of its hysteresis performance.

Two additional configurations (iterations 17–18) replaced the beam material with metal and an alternative polymer, respectively, while retaining the ring-based mounting concept. Static screening revealed excessive hysteresis and mechanical memory effects in the metal variant, attributable to its stiffness mismatch with the shaft, and poor repeatability in the polymer variant; both were therefore abandoned in favor of the PETG geometries and are not analyzed further.

**Figure 19:**
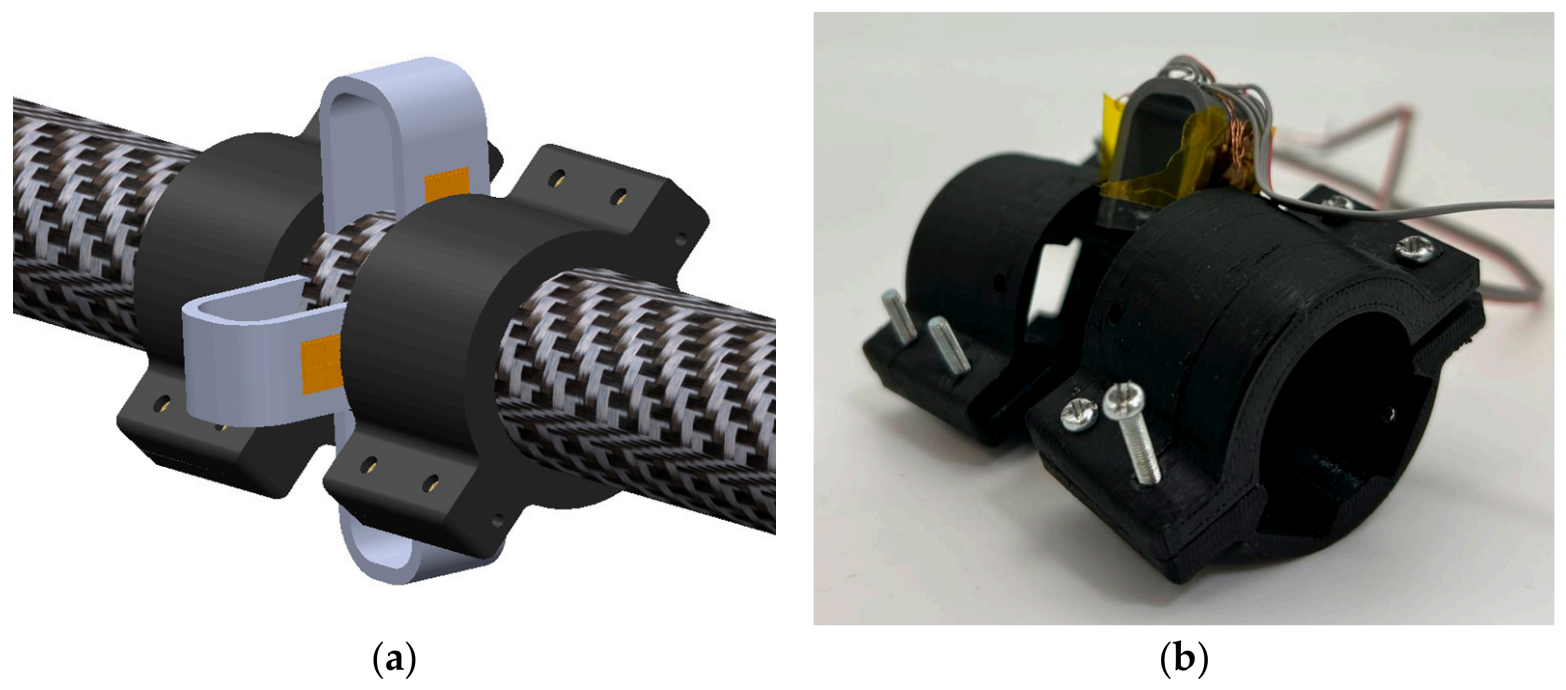
Inverted U-shaped beam configuration (iteration 19): (**a**) CAD Model; (**b**) real prototype.

Iteration 19 introduced an inverted U-shaped beam geometry to modify the structural load path and investigate whether sensor placement is related to the load application point, rather than to the beam cross-section geometry alone, as the governing parameter for peak-force fidelity. The inverted U-shaped beam (Figure 19) was evaluated using the same static protocol as previous iterations, with a bonded strain gauge on the shaft as the force reference. Two strain gauges were embedded symmetrically in the shell structure, one on the left and one on the right side of the beam. To investigate the influence of sensor position on measurement quality, the system was also tested in an inverted configuration, rotating the assembly to alter the relative position of each gauge with respect to the load application point.

**Figure 20:**
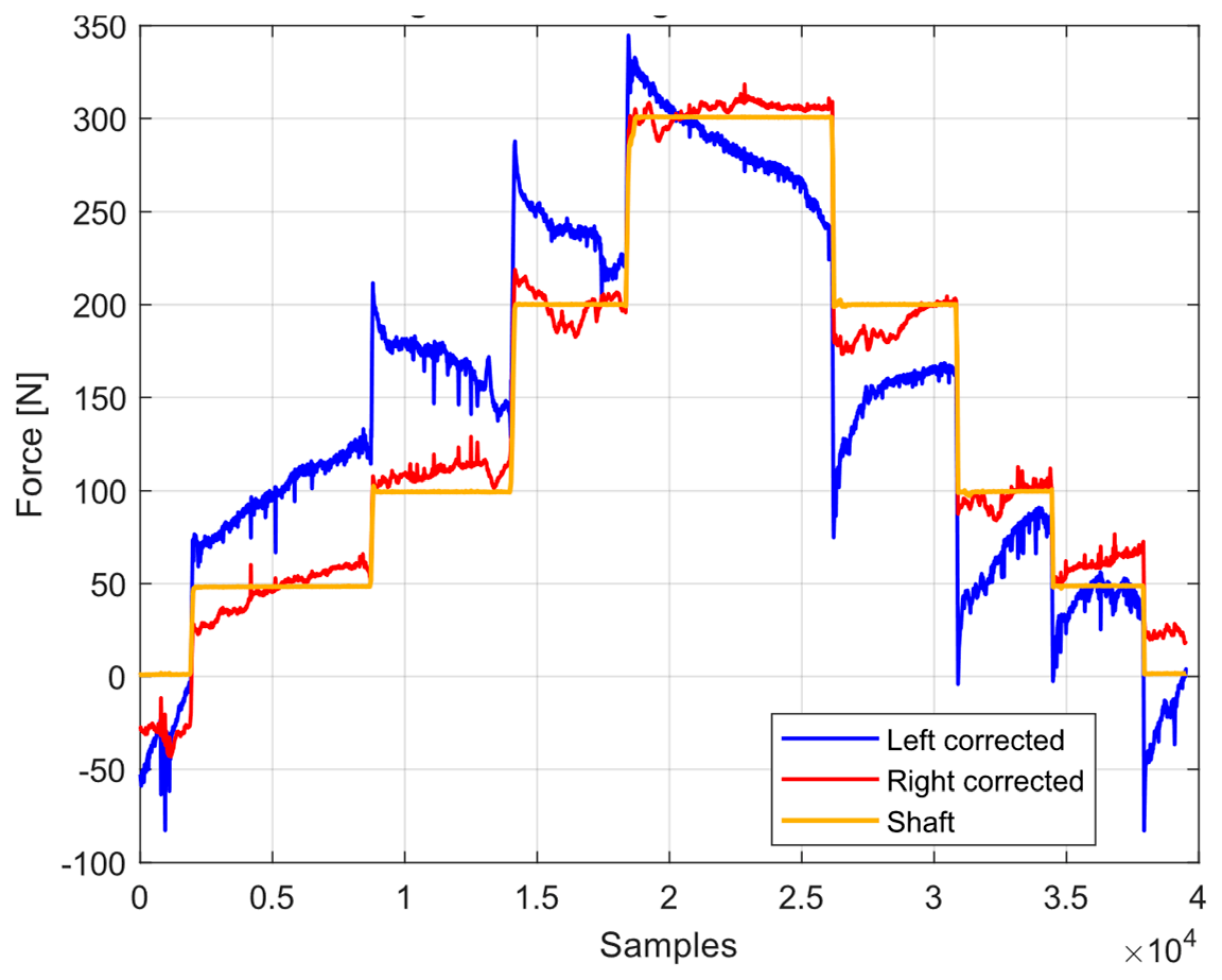
Force signal comparison for iteration 19 after magnitude correction: shaft reference (yellow), left gauge corrected (blue), and corrected right gauge (red), showing the overall agreement and residual transient fluctuations under the tested static loading conditions.

Under the tested mounting and loading conditions, the inverted U-shaped beam approximated the overall response of the reference shaft, with correspondence in curve shape and temporal response under both static (Figure 20) and dynamic loading. Force peaks and the temporal sequence of loading events were identified by both embedded gauges, indicating that the system retained the main temporal features of the applied load in these tests.

Figure 20 also shows transient fluctuations following some load changes and gradual signal variation during selected load plateaus, indicating that magnitude correction did not eliminate all time-dependent instability. These effects may arise from interface settling, micro-slip, polymer viscoelasticity, or variability in load application; however, the present protocol was not designed to isolate these mechanisms or to characterize creep independently. The distal sensing position was therefore selected as the best-performing condition among those evaluated rather than as a configuration free from transient measurement artifacts.

As shown in Table 9, systematic differences were observed between the two gauges in their positions relative to the load application point. In the normal configuration, the distal gauge (right) achieved R^2^ = 0.990, MAE = 7.78 N, and hysteresis of 3.4% of the applied load range, the lowest hysteresis of any shell configuration evaluated in this study, while the proximal gauge (left) showed substantially poorer performance, with R^2^ = 0.884, MAE = 27.73 N, and hysteresis of 25.1%. Notably, calibration failed to improve the proximal gauge (MAE before/after: 25.7/27.7 N), confirming that its error is dominated by hysteresis rather than gain and is therefore not correctable by linear calibration. The markedly elevated hysteresis of the proximal gauge is consistent with local stress-concentration effects near the load application zone, which promote micro-slip and deformation accumulation at the interface.

Critically, the inversion experiment confirmed that this behavior is positional rather than sensor-specific. When the assembly was rotated, the left gauge, now distal, improved markedly (R^2^ = 0.954, MAE = 17.68 N, hysteresis of 9.1%), while the right gauge, now proximal, degraded in every metric (R^2^ = 0.963, MAE = 16.29 N, hysteresis of 9.3%, versus 0.990, 7.78 N, and 3.4% when distal). The response pattern shifted consistently with position, demonstrating that measurement quality is governed by the gauge’s distance from the load application point rather than by any intrinsic property of the sensor. It is also worth noting that both gauges performed more similarly in the inverted configuration, suggesting that the inverted orientation distributes the structural load more uniformly between the two sensing locations.

The inversion experiment indicated a positional effect because each gauge showed improved performance when located distally rather than proximally, although the distal position did not outperform the proximal position in every metric within the inverted configuration (Table 9). The residual hysteresis—minimal at the distal position (3.4% of the applied load range), but substantial at the proximal position (up to 25.1%)—requires contextualization relative to the temporal structure of the paddle stroke, which comprises a biomechanically active water phase, during which force rises from zero to peak and returns, followed by an aerial recovery phase of negligible applied force [3,4,13]. Hysteresis in the present configuration manifests predominantly at low force levels during unloading, consistent with micro-slip at reduced contact forces [19,20], and is therefore expected to concentrate at the phase transition rather than within the peak-force region. For applications centered on the water phase, including peak force quantification, force–time profiling, and inter-limb asymmetry analysis [2,3], the distal gauge provided the closest agreement with the reference among the evaluated sensing positions; for applications requiring accuracy across the full stroke cycle, including recovery, the elevated hysteresis of the proximal gauge is a substantive limitation, and the distal position should be considered the only viable sensing location. Taken together, the highest R^2^, the lowest MAE, and a well-defined structural load path identify the distal sensing position of the inverted U-shaped beam as the best-performing measurement location among those evaluated under the present conditions, provided that per-session zeroing is applied. Although the inverted U-shaped beam did not provide uniform measurement performance across both sensing positions, its distal sensing position showed the closest agreement with the reference among the configurations evaluated. The inverted U-shaped geometry with distal gauge placement was therefore selected as the basis for subsequent prototype development. This result applies specifically to the distal sensing position; the proximal gauge continues to exhibit high MAE and hysteresis and therefore does not share the performance improvement observed at the distal location.

## 4. Synthesis of Design Evolution and Final Design Selection

The experimental results across the nineteen design iterations indicated that strain transmission through a removable interface is influenced by a coupled interaction among geometry, material compliance, contact conditions, structural stiffness, boundary constraints, and fixation strategy. The decision matrix presented in Table 1 summarizes the experimental evidence and criteria that guided the transition between design concepts.

Across the iterative process, each phase addressed the dominant limitation identified in its predecessor while exposing a new trade-off: compliance was exchanged for rigidity, partial contact for circumferential enclosure, and distributed contact for a defined structural load path. The on-water analysis of Phase IV quantified the cost of contact-driven transmission directly, temporal drift near the hinge, and installation-dependent gain variability near the screw closure, and the beam-based configurations of Phase V progressively removed the measurement’s dependence on contact uniformity, with beam cross-section (Table 6) and boundary conditions (Table 4) emerging as the dominant parameters.

Although these principles may inform the development of other removable sensing interfaces on cylindrical substrates, their applicability beyond the tested paddle-shaft configuration requires validation under different geometries, materials, loading conditions, and environmental conditions. First, adequate sensor–interface integrity must be established before the influence of bulk-material compliance on strain transmission can be quantified. Second, circumferential contact improves global tracking but introduces sensitivity to local discontinuities that cannot be resolved solely through clamping optimization. Third, fixation strategy and boundary conditions are primary determinants of repeatability, independent of overall geometry. Fourth, sensing elements must be positioned remote from geometric discontinuities and load application zones to avoid locally perturbed deformation fields. Fifth, concentrating deformation within a defined structural element, rather than relying on distributed contact, provides a more predictable and controllable measurement pathway. Collectively, these principles provide testable design hypotheses for future removable sensing interfaces on cylindrical substrates, rather than universally validated rules.

The distal sensing position of the inverted U-shaped beam configuration (iteration 19) was selected as the basis for subsequent development because it achieved the highest linearity and the lowest absolute error and hysteresis among the evaluated shell measurement locations (Table 9). The inversion experiment further indicated that the observed performance differences were associated with the gauges’ positions relative to the load application zone rather than with the identity of the two gauges. However, the substantially poorer response of the proximal gauge demonstrates that the inverted U-shaped geometry does not provide uniformly acceptable measurement performance across all sensing locations. This selection must nonetheless be read against the trade-offs that governed the entire development process: no configuration eliminated all sources of measurement uncertainty simultaneously. The straight beam (iteration 16) performed consistently across the load cycle but introduced systematic error at peak force, i.e., the biomechanically critical region, as revealed by SPM analysis; the distal sensing position of the inverted U-shaped beam showed the closest peak-force agreement, whereas substantial residual hysteresis remained at the proximal position and during the low-force unloading phase. The decisive result was therefore the identification of a sensing position that provided the most favorable balance among linearity, absolute error, hysteresis, and peak-force fidelity within the configurations evaluated. Direct metric-level comparison with existing removable systems is constrained by heterogeneous reporting: Sturm et al. [15] report recalibration-dependent error, and Guseinov et al. [16] report correlation coefficients alongside qualitative observations, whereas the present study reports R^2^ and MAE in (N), reflecting the availability of a calibrated force reference absent from both prior works. Two observations nonetheless align the present work with the existing literature: the installation-dependent gain variability identified in both prior studies as a fundamental limitation of removable systems is substantially reduced in the beam-based configurations developed here, where the structural load path is more controlled. The low-force nonlinearity reported by Guseinov et al. [16] is consistent with the micro-slip behavior characterized throughout this study, suggesting a common mechanical origin that the beam-based approach directly addresses.

## 5. Conclusions

Within the configurations and loading conditions evaluated, this study showed that the measurement response of a removable strain-sensing interface is strongly influenced by the structural load path, sensor placement, and fixation strategy rather than by geometric conformity alone. A systematic investigation of nineteen interface configurations was conducted across five conceptual phases, providing design indications for the development of non-invasive sensing interfaces for paddle shafts.

Within the compliant materials and fabrication approaches evaluated, the interfaces were unsuitable for systematic strain measurement because sensor–material detachment and manufacturing defects prevented reliable data collection. Rigid shell geometries substantially improved strain transmission, but circumferential contact alone was insufficient; local contact nonuniformities and geometric discontinuities near closure mechanisms introduced location-dependent errors that persisted across all shell configurations. Under the experimental conditions used in this study, the transition to beam-based geometries, culminating in the inverted U-shaped beam, indicated that concentrating deformation within a defined structural element can provide a more controlled measurement pathway than relying on distributed contact. The inversion experiment supported the conclusion that sensor placement relative to the load application zone influences measurement quality independently of the identity of the two gauges used, providing a design indication for future sensing configurations. Cross-session calibration errors remained substantial, with mean values of approximately 14% for the straight beam and 20% for the curved beam. In this context, cross-session errors in the 14–20% range are adequate for monitoring of relative trends or load progression, but they constrain applications that require accurate absolute force quantification unless recalibration is performed. Thus, although the straight beam improved calibration stability, the present results do not support calibration-free absolute force measurement across sessions, and recalibration remains necessary when accurate force magnitude is required.

The iterative process documented here also yields a methodological lesson that extends beyond the specific application. In the design of removable sensing interfaces, no single configuration is likely to eliminate all trade-offs simultaneously, and the search for a globally optimal solution is less productive than the systematic identification of which trade-offs are acceptable given the intended measurement context. In the present case, the biomechanical specificity of paddle-based water sports, force concentrated in the water phase (negligible during recovery) defined the acceptability boundary that guided the final selection. The proposed design framework may provide a methodological basis for the development of non-invasive sensing interfaces for other cylindrical structural systems. However, its transferability must be evaluated using application-specific geometries, materials, fixation conditions, loading profiles, and environmental conditions. The findings should be interpreted within the scope of the experimental conditions evaluated. The influence of support conditions was investigated only during the exploratory evaluation of the Phase II half-shell (Section 3.2, Figure 7). The full wrap-around shell developed in Phase III, the hinge-and-dual-closure shell developed in Phase IV, and the subsequent beam-based prototypes of Phase V were characterized in the laboratory using the standard static configuration (Figure 2). No additional experiments were conducted to determine whether these configurations were similarly sensitive to changes in shaft support conditions. Consequently, the reported comparisons apply to the standardized boundary conditions used in this study, and support-condition sensitivity cannot be generalized across all development phases. Laboratory characterization was conducted using the shaft geometry, materials, loading ranges, and installation procedures described in this study, while the exploratory on-water assessment involved a single athlete. The effects of shaft diameter and material, inter-athlete variability, repeated installation by different operators, temperature, humidity, and water exposure were not systematically evaluated. Consequently, the present results do not establish measurement reliability across different athletes, shafts, or environmental conditions. Further validation is required before the interface can be considered suitable for routine training or performance-monitoring applications.

## 6. Future Developments and Research Directions

Several directions for further development emerge from the findings of this study. The residual positional dependence of the strain gauge response identified in iteration 19 motivates a systematic investigation of optimal sensor placement relative to the load application point, informed by the paddle stroke biomechanics literature. Expanding the sensing configuration to four gauges will enable simultaneous measurements across multiple planes, improving the correspondence between the shell response and the shaft’s true mechanical behavior under realistic paddling loads. From a mechanical standpoint, localized locking features or surface treatments at the shell–shaft interface could reduce relative displacement and micro-slip under cyclic loading, addressing the residual hysteresis observed across configurations, and the development of analytical or numerical models of deformation transfer through beam-based interfaces would enable model-based correction of systematic errors, reducing the dependence on empirical calibration. Finally, validation should be extended to athletes with different anthropometric and technical characteristics; paddle shafts with different diameters, materials, and stiffness properties; and repeated installation sessions conducted by different operators. The influence of temperature, humidity, and water exposure on zero stability, calibration, hysteresis, and repeatability should also be quantified. These steps, together with integration into a compact wireless data-acquisition system, are required before the platform can be considered suitable for routine training and performance-monitoring applications.

## 7. Patents

A provisional patent application covering the removable shell interface described in this manuscript was filed with the Portuguese Institute of Industrial Property (INPI) under application no. 20262008187943 on 29 July 2026.

## Figures and Tables

**Table 1:** Decision matrix summarizing the experimental evidence and criteria supporting the main decisions in the iterative development process.

Development Decision	Experimental Evidence and Variables Explored	Limitation Classification	Decision Criterion	Design Consequence
Phase I to Phase II	Visual inspection revealed delamination or detachment near the embedded sensors. Adjustments to the TPU printing conditions and the use of two compliant sensor-encapsulation approaches did not provide specimens suitable for systematic quantitative testing.	Structural within the tested sensor-integration strategy	Sensor-interface integrity could not be maintained using the tested embedded-gauge approaches.	Adopt a rigid interface with a surface-mounted strain gauge.
Phase II to Phase III	R^2^ remained between 0.48 and 0.54 under hand-held, table-supported, and bearing-supported conditions, and the dynamic response was strongly attenuated.	Structural	Partial circumferential contact remained sensitive to boundary conditions and did not adequately transmit the dynamic response.	Replace the half-shell with full circumferential enclosure.
Phase III to Phase IV	The external clamp increased R^2^ from 0.41 to 0.97, but hysteresis remained 34.21 µε, compared with 3.41 µε for the bonded reference. Configurations without a clamp and with one clamp were evaluated.	Structural within the single-point clamping strategy	Single-point clamping improved linearity but did not sufficiently reduce hysteresis.	Introduce a continuous hinge and two closure points.
Phase IV to Phase V	The dual-closure shell preserved the cyclic signal, but drift, hysteresis accumulation, and sensing-position-dependent behavior remained under static, dynamic, and exploratory on-water conditions.	Structural	Measurement performance remained dependent on local contact and closure conditions despite the distributed clamping strategy.	Replace distributed-contact sensing with a beam-based load-transfer pathway.
Iterations 7–14 to Iterations 157–16	Changes to ring position and width, free-end conditions, adhesive layers, beam width, and bridge configuration altered the balance between linearity and hysteresis, but no ring-based configuration improved both simultaneously.	Structural within the ring-mounted concept	The trade-off between linearity and hysteresis persisted across the tested ring-based variants and was associated with discrete fixation constraints.	Eliminate the rings and integrate the beam into the enclosing structure.
Iteration 15 to Iteration 16	The straight beam reduced MAE, RMSE, CV, and hysteresis relative to the curved beam while retaining the same shell-integrated load-transfer concept.	Parametric/geometric	Performance improved after changing the beam cross-section without changing the underlying structural concept.	Retain the shell-integrated concept and continue development using the straight beam.
Iterations 167–18 to Iteration 19	The straight beam retained differences in the peak-force region, while the alternative beam materials evaluated in iterations 177–18 did not resolve the observed hysteresis or repeatability limitations.	Structural/positional	The remaining errors indicated that the load path and sensor position relative to the load application zone required further investigation.	Introduce the inverted U-shaped geometry and use an inversion test to isolate sensor-position effects.
Selection within Iteration 19	Measurement performance changed when the assembly was inverted. The distal sensing position presented the most favorable combination of R^2^, MAE, RMSE, and hysteresis.	Parametric within the selected geometry	Performance varied with sensing position relative to the load application zone rather than with the identity of the two gauges.	Select the distal sensing position as the basis for subsequent prototype development.

Note: The classifications presented in this matrix describe the operational decision process used during exploratory development and should not be interpreted as predefined quantitative acceptance thresholds. TPU—thermoplastic polyurethane; R^2^—coefficient of determination; µε—microstrain; MAE—mean absolute error; RMSE—root mean square error; CV—coefficient of variation.

**Table 2:** Experimental provenance by design phase: acquisition system, bridge configuration, force reference, and performance metrics reported.

Phase (Iterations)	Acquisition System	Bridge Configuration	Force Reference	Metrics Reported
I (1–2)	V-Link, 256 Hz	Quarter-bridge	Bonded strain gauge (shaft)	Qualitative feasibility (sensor adhesion, geometric conformity)
II (3)				Pearson r, slope, offset, R^2^
III (4–5)				R^2^, hysteresis (µε)
IV (6)				R^2^, hysteresis (µε), MAE, and RMSE; on-water: slopeRes, |ΔMid|, R, and S
V (7–11)	Custom PCB, 70 Hz	Quarter-bridge	Bonded strain gauge (shaft)	R^2^, hysteresis (raw ADC units), CV, FSO
V (12–14)		Full-bridge		R^2^, hysteresis (raw ADC units), CV, FSO
V (15–16)			Calibrated load cell	R^2^, hysteresis (% load range), CV, MAE, RMSE; SPM
V (17–18)		Quarter-bridge	Bonded strain gauge (shaft)	Qualitative screening; discontinued (Section 3.5)
V (19)		Full-bridge		R^2^, hysteresis (% load range), MAE, RMSE

r—Pearson correlation coefficient; R^2^—coefficient of determination; µε—microstrain; slopeRes—residual slope of the signal over each repetition (µε/s); |ΔMid|—absolute mid-point offset (µε); R—proportionality ratio between shell and reference peak amplitudes; S—symmetry index between opposing channels; ADC—analog-to-digital converter; CV—coefficient of variation; FSO—full-scale output error; MAE—mean absolute error (N); RMSE—root mean square error (N); SPM—Statistical Parametric Mapping.

**Table 3:** Quantitative assessment of the half-shell response under three boundary conditions: Pearson correlation, slope, offset, and R^2^.

Condition	Pearson Correlation	Slope	Offset (µε)	Linearity (R^2^)
Hand-held	0.74	3.14	47.93	0.54
Table-supported	0.69	2.43	33.71	0.48
Bearing-supported	0.71	3.70	−14.24	0.51

**Table 4:** Static performance of full wrap-around shell variants compared to the bonded reference: linearity (R^2^) and hysteresis (µε).

Condition	Linearity (R^2^)	Hysteresis (µε)
Bonded reference	0.99	3.41
Simple	0.41	37.93
With Clamps	0.97	34.21

**Table 5:** Static performance of the hinge-and-dual-closure shell (iteration 6) relative to the corresponding bonded shaft gauges over the 0–200 N loading range (single static trial, shown in Figure 12a).

Sensing Location	Measurement	R^2^	Hysteresis (µε)	MAE (N)	RMSE (N)
Top	Shell gauge	0.998	16.80	2.26	2.47
Top	Bonded reference	1.000	3.54	0.52	0.56
Bottom	Shell gauge	0.484	329.75	37.23	43.75
Bottom	Bonded reference	0.997	25.24	3.23	3.36

**Table 6:** Quantitative performance of beam-based configurations (iterations 7–14): linearity (R^2^), hysteresis (raw ADC units), CV (%), and FSO (%). Cell shading indicates relative performance within each metric row (darker = better).

Iteration (n = 4)	7	8	9	10	11	12	13	14
Linearity (R^2^)	0.879 ± 0.153	0.697 ± 0.220	0.440 ± 0.032	0.586 ± 0.261	0.528 ± 0.333	0.709 ± 0.099	0.881 ± 0.119	0.904 ± 0.067
Hysteresis (ADC units)	26 614	84 554	8 786	10 914	6 729	18 283	60 525	53 202
CV mean (%)	0.6–2.2	0.2–4.2	0.2–0.4	0.3–0.6	0.3–0.6	0.3–1.0	1.82–8.45	1.36–7.49
FSO mean (%)	20	42	65	49	61	38	22	19

CV—coefficient of variation of the mean signal; FSO—full-scale output error.

**Table 7:** Static performance of the shell-integrated beam configurations (iterations 15–16) and bonded reference (REF; see Section 2): CV, hysteresis (% of applied load range, peak level excluded), MAE, and RMSE. Values are the means of two loading trials; ± indicates the half-range for MAE/RMSE and the SD across load levels for CV and hysteresis. REF calibrations are session-to-session.

Shell Design	CV (%)	Hysteresis (%)	MAE (N)	RMSE (N)
PETG—curved beam	3.70 ± 2.60	13.70 ± 1.30	17.02 ± 0.63	18.66 ± 0.54
REF	0.80 ± 1.00	0.40 ± 0.40	0.74 ± 0.39	0.79 ± 0.41
PETG—straight beam	0.50 ± 0.80	9.80 ± 1.70	13.05 ± 0.09	14.52 ± 0.09
REF	0.02 ± 0.02	0.08 ± 0.05	1.21 ± 0.00	1.46 ± 0.00

CV—coefficient of variation of the mean signal; MAE—mean absolute error; RMSE—root mean squared error.

**Table 8:** Cross-session calibration characterization for iterations 15–16: maximum error (N) and mean percentage error over the 50–300 N load levels. Shell values are the mean ± half-range of two independent loading trials evaluated with the calibration line obtained in the reference session; REF rows report the bonded gauge evaluated with its own session calibration, establishing the within-session error floor of the measurement chain.

	Straight Beam		Curved Beam	
	REF	Test	REF	Test
Error Max (N)	2.88 ± 0.00	25.47 ± 0.10	1.17 ± 0.72	25.41 ± 0.20
Error Mean (%)	1.1 ± 0.01	14.20 ± 0.41	0.80 ± 0.65	20.2 ± 0.26

**Table 9:** Static performance metrics for the inverted U-shaped beam (iteration 19) under normal and inverted configurations: R^2^, MAE after calibration (N), RMSE after calibration (N), and hysteresis (%) for each gauge position. Cell shading indicates relative performance within each metric column (darker = better).

Configuration	Sensor	R^2^	MAE (N)	RMSE (N)	Hysteresis (%)
Normal	Right (distal)	0.990	7.78	9.28	3.4
Normal	Left (proximal)	0.884	27.73	33.38	25.1
Inverted	Left (distal)	0.954	17.68	21.37	9.1
Inverted	Right (proximal)	0.963	16.29	18.96	9.3
Reference (shaft)	—	1.000	—	—	0.6/0.2

MAE—mean absolute error; RMSE—root mean squared error.

## Data Availability

The data presented in this study are available on request from the corresponding author.

## Appendix A

### Appendix A.1

The four cycle-level metrics used in the on-water evaluation of Phase IV (Section 3.4) are formally defined in Table A1. All metrics were computed cycle-by-cycle for each sensing channel, and their distributions were characterized using the median and interquartile range (IQR), given the cycle-by-cycle variability and the limited number of repetitions per condition, which preclude assumptions of normality.

**Table A1:** Formal definitions of the on-water measurement quality metrics: residual slope (slopeRes), absolute mid-point offset (|ΔMid|), proportionality ratio (R), and symmetry index (S).

Metric	Definition	Units
slopeRes	Residual slope of the signal over each test repetition, computed as the linear trend coefficient by least-squares regression; positive values indicate progressive drift and negative values indicate relaxation.	µε/s
|ΔMid|	Absolute difference between the signal baseline at the beginning and at the midpoint of each repetition, reflecting asymmetry between the loading and unloading phases.	µε
R	Ratio of the peak amplitudes of the shell and reference signals within each stroke cycle; values close to unity indicate a proportional response.	–
S	Ratio of the signal amplitudes of the opposing sensing channels; a value of unity indicates perfect symmetry.	–
